# Essential Oils of Four Virginia Mountain Mint (*Pycnanthemum virginianum*) Varieties Grown in North Alabama

**DOI:** 10.3390/plants10071397

**Published:** 2021-07-08

**Authors:** William N. Setzer, Lam Duong, Trang Pham, Ambika Poudel, Cuong Nguyen, Srinivasa Rao Mentreddy

**Affiliations:** 1 Department of Chemistry, University of Alabama in Huntsville, Huntsville, AL 35899, USA; cvn0001@uah.edu; 2 Aromatic Plant Research Center, 230 N 1200 E, Suite 100, Lehi, UT 84043, USA; apoudel@aromaticplant.org; 3 Department of Biological and Environmental Sciences, Alabama A & M University, Normal, AL 35762, USA; lduong@bulldogs.aamu.edu (L.D.); trang.pham@bulldogs.aamu.edu (T.P.)

**Keywords:** pulegone, isomenthone, menthone, thymol, *p*-cymene, chemotypes, seasonal variation, enantiomeric distribution

## Abstract

Virginia mountain mint (*Pycnanthemum virginianum*) is a peppermint-flavored aromatic herb of the Lamiaceae and is mainly used for culinary, medicinal, aromatic, and ornamental purposes. North Alabama’s climate is conducive to growing mint for essential oils used in culinary, confectionery, and medicinal purposes. There is, however, a need for varieties of *P. virginianum* that can be adapted and easily grown for production in North Alabama. Towards this end, four field-grown varieties with three harvesting times (M1H1, M1H2, M1H3; M2H1, M2H2, M2H3; M3H1, M3H2, M3H3, M4H1, M4H2, M4H3) were evaluated for relative differences in essential oil yield and composition. Thirty-day-old greenhouse-grown plants of the four varieties were transplanted on raised beds in the field at the Alabama A & M University Research Station in North Alabama. The plots were arranged in a randomized complete block with three replications. The study’s objective was to compare the four varieties for essential oil yield and their composition at three harvest times, 135, 155, and 170 days after planting (DAP). Essential oils were obtained by hydrodistillation with continuous extraction with dichloromethane using a Likens–Nickerson apparatus and analyzed by gas chromatographic techniques. At the first harvest, the essential oil yield of the four varieties showed that M1H1 had a yield of 1.15%, higher than M2H1, M3H1, and M4H1 with 0.91, 0.76, and 1.03%, respectively. The isomenthone concentrations increased dramatically through the season in M1 (M1H1, M1H2, M1H3) by 19.93, 54.7, and 69.31%, and M3 (M3H1, M3H2, M3H3) by 1.81, 48.02, and 65.83%, respectively. However, it increased only slightly in M2 and M4. The thymol concentration decreased slightly but not significantly in all four varieties; the thymol in M2 and M4 was very high compared with M1 and M3. The study showed that mountain mint offers potential for production in North Alabama. Two varieties, M1 and M3, merit further studies to determine yield stability, essential oil yield, composition, and cultivation development practices.

## 1. Introduction

Discovered and named ‘mountain mint’ by the French Botanist Andre Micaux [1], *Pycnanthemum* Michx. is an herbaceous perennial belonging to the family Lamiaceae. The plants can grow up to 1 meter in height with delicate, angular stems and 4–6 cm long narrowly lanceolate leaves. The leaves are known for their mild mint flavor. The plants grow well in semi-shaded woodlands and along waterways on well-drained, light, sandy, loam clay soils with a pH ranging from slightly acidic to slightly alkaline [2].

*Pycnanthemum* Michx. has been reported to consist of an estimated 20 species according to World Flora Online [3], all of which occur in North America: ***Pycnanthemum albescens* Torr. & A. Gray**, *Pycnanthemum beadlei* (Small) Fernald, *Pycnanthemum californicum* Torr. ex Durand, *Pycnanthemum clinopodioides* Torr. & A. Gray, ***Pycnanthemum curvipes* (Greene) E.Grant & Epling**, ***Pycnanthemum flexuosum* (Walter) Britton, Sterns & Poggenb.**, *Pycnanthemum floridanum* E.Grant & Epling, ***Pycnanthemum incanum* (L.) Michx.**, ***Pycnanthemum loomisii* Nutt.**, *Pycnanthemum monotrichum* Fernald, *Pycnanthemum montanum* Michx., ***Pycnanthemum muticum* (Michx.) Pers.**, ***Pycnanthemum nudum* Nutt.**, ***Pycnanthemum pycnanthemoides* (Leavenw.) Fernald**, *Pycnanthemum setosum* Nutt., ***Pycnanthemum tenuifolium* Schrad.**, *Pycnanthemum torreyi* Benth., ***Pycnanthemum verticillatum* (Michx.) Pers.**, ***Pycnanthemum virginianum* (L.) T. Durand & B.D. Jacks. ex B.L. Rob. & Fernald**, and *Pycnanthemum viridifolium* E.Grant & Epling. The species highlighted in **bold** have been recorded in the state of Alabama. Nearly 50% of the known species are found in Alabama, which underscores the adaptation of mountain mint to Alabama environments.

Several *Pycnanthemum* species have been used in Native American traditional medicine [1]. The Choctaw took a hot decoction of *P. albescens* leaves as a diaphoretic for colds. The Miwok used *P. californicum* as a treatment for colds. A poultice of leaves of *P. flexuosum* or *P. incanum* was used by the Cherokee to relieve headache, while a leaf infusion of these plants was taken for fevers. The Lakota took an infusion of the leaves of *P. virginianum* for coughs. In addition, *P. flexuosum* and *P. incanum* were used by the Cherokee for food, and the Chippewa used *P. virginianum* to season meat or broth. Today, mountain mint is popularly used as a mild-flavored tea, and the leaves and buds are often eaten in salads. Mountain mint tea is known to be curative, diaphoretic, and carminative [1,2]. The tea made of mountain mint leaves is used for treating menstrual disorders, mild headaches, fevers, colds, coughs, and indigestion [1,2,3]. Mountain mint has been shown to cause abortions if consumed by pregnant women [1].

The essential oil composition of mountain mints varies considerably among species, and the major components are carvacrol, menthone, isomenthone, β-elemene, limonene, piperitone (minty and camphor-like odor [4]), and germacrene D, characteristic of species in the Lamiaceae [5,6]. The uses of *Pycnanthemum* species are based on essential oil composition, e.g., *P. virginianum* rich in menthone and isomenthone have culinary and medicinal uses; *Pycnanthemum* species rich in β-elemene and low in pulegone are known to attract beneficial insects, mainly bees and butterflies, whereas species such as *P. muticum*, rich in pulegone, are insect repellants. If consumed, pulegone can be toxic to the liver, but it is apparently safe to rub *P. muticum* herb on clothes to deter chiggers, gnats, and ticks [7]. Species with little or no pulegone are used for making teas and infusions. The global market demand for mint essential oil was an estimated 177.88 million USD in 2018 with a predicted annual growth rate of 9.2% between 2014 and 2025 [8]. Thus, mountain mint rich in menthone and isomenthone may be suitable for pharmaceutical and nutraceutical, food & beverage, cosmetic, aromatherapy, and cleaning products markets [8]. About 50% of mountain mint species grow wild in Alabama and are adapted to shady environments and sandy marginal soils. Therefore, this crop merits consideration for evaluation as an alternative, niche-market cash crop in Alabama. Towards this end, four varieties of *P. virginianum* were evaluated for their leaf biomass and essential oil content and composition.

## 2. Results and Discussion

### 2.1. Fresh Leaf Biomass Yield

**Figure 1:**
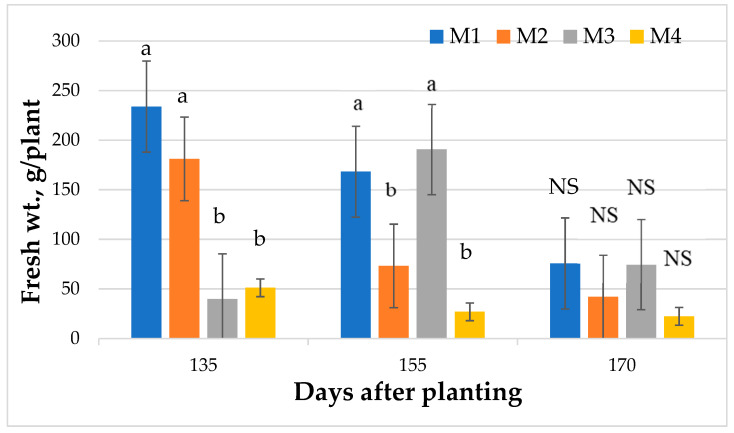
Changes with time in leaf fresh biomass of four mountain mint varieties grown in North Alabama, USA. Bars with same letter are not significantly different at *p* ≤ 0.05. NS = not significant.

There were significant differences among varieties for fresh leaf biomass at 135 and 155 days after transplanting (DAP), but no such differences existed at 179 DAP (Figure 1). At 135 DAP, M1 and M2 had significantly more fresh leaf biomass than other varieties. At 155 DAP, Varieties M1 and M3 had similar leaf fresh weights and were significantly greater than that of M2 and M4. At 170 DAP, however, all varieties produced similar fresh leaf biomass. The fresh leaf biomass of all varieties decreased with age, and at 170 DAP, it was less than half of that produced at 135 DAP. M3 did not fare well at the first time of planting because the plants in two replications died, but this may be related to inadequate propagules and the harvesting plan. July, the month following planting, received the lowest amount of precipitation combined with high temperatures (see Supplementary Figure S1), which may have affected the poorly established plants of M3. However, this variety has shown consistently good growth in the greenhouse and in the field trials in progress.

**Figure 2:**
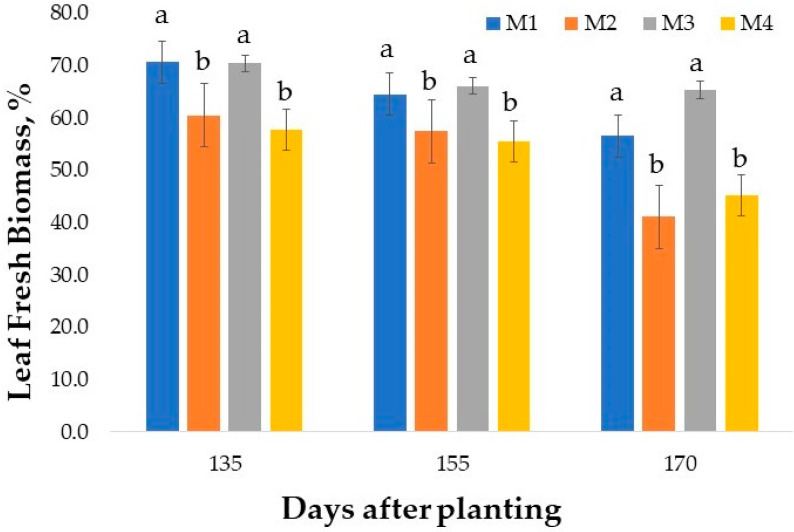
Variation among four mountain mint varieties for fresh leaf biomass as a percentage of whole plant biomass at three times of harvest. Alabama, USA. Bars with same letter are not significantly different at *p* ≤ 0.05.

The fresh leaf biomass as a percentage of whole plant biomass varied with variety (Figure 2). At the first time of harvest (135 DAP), varieties M1 and M3 with leaf biomass of nearly 70% of the whole plant biomass were superior in leaf production than M1 and M2. At 155 DAP, all varieties had lower levels of leaf biomass as a percentage of whole-plant biomass with narrow differences between varieties. However, M3 had a higher percentage of leaf biomass than M4. At 170 DAP, the leaf biomass as a percentage of the whole plant was lower among all varieties relative to those at 155 or 135 DAP; the magnitude of differences between varieties increased. Thus, M3 partitioned a greater percentage of leaves compared to other varieties. M1 with 58% was superior to M2 and M4, which had a similar percentage of leaf biomass. In general, the M1 and M3 were consistently superior to M2 and M4. Seasonal effects were observed as all varieties had lower leaf biomass as the season advanced. Although the literature on the growth and yield of mountain mint is scarce, there is published research on variation in biomass accumulation and oil content in species belonging to the Lamiaceae [9,10,11,12]. Brar et al. [10] reported a larger leaf-to-stem ratio in *Mentha arvensis* L. Higher accumulations of fresh leaf biomass during the mid-season and decline as the crop matured has been observed in *Mentha piperita* L. [12] as well as *M. arvensis* [11], a trend observed in this study.

There were limited time-of-harvest × variety interactions for fresh leaf biomass. The interaction of M1 was significant with the first and second time of harvest, whereas the interaction of M2 and M3 was significant with the first and second time of harvest, respectively. All other interactions among time of harvest and varieties were not significantly different at *p* ≤ 0.05.

### 2.2. Chemical Composition of Essential Oils

The chemical compositions of the *P. virginianum* essential oils have been determined by gas chromatography–mass spectrometry (GC-MS) and quantified by gas chromatography–flame ionization detection (GC-FID). The essential oil compositions are compiled in Supplementary Tables S1–S4. The major components of the four varieties of *P. virginianum* over three harvest dates are summarized in Table 1.

**Figure 3:**
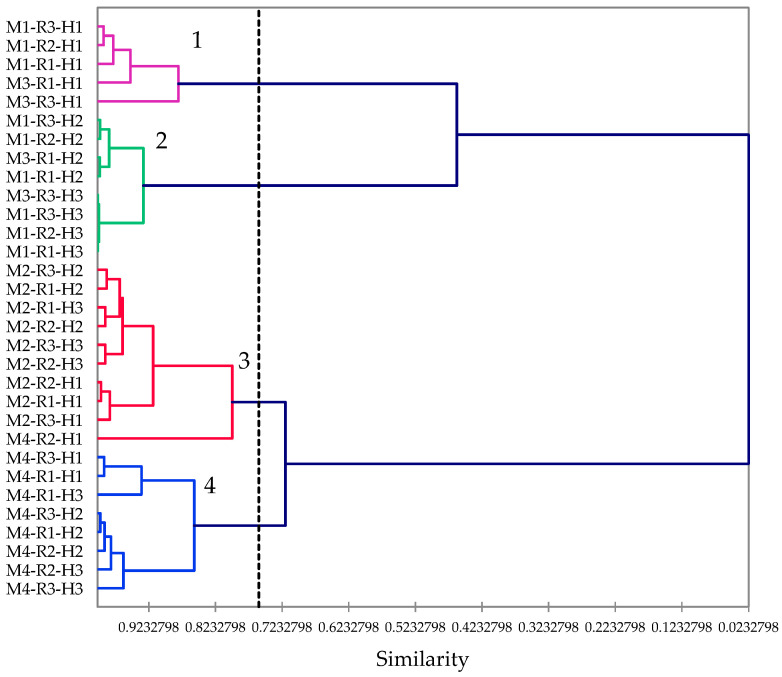
Dendrogram obtained by hierarchical cluster analysis of the 16 most abundant components of *Pycnanthemum virginianum* essential oils. ‘M’ represents the varieties, ‘R’ is the replicate of each variety, and ‘H’ is the harvesting times.

The four varieties of *P. virginianum* showed different volatile chemical profiles and apparently define different chemical variations of this species. In order to discern the phytochemical differences between these varieties, a hierarchical cluster analysis was carried out using 16 of the most abundant essential oil components (1-octen-3-ol, myrcene, *p*-cymene, limonene, γ-terpinene, *cis*-sabinene hydrate, menthone, isomenthone, *trans*-isopulegone, *cis*-piperitenol, pulegone, thymol, carvacrol, unidentified (RI 1345), (*E*)-β-caryophyllene, and germacrene D) (Figure 3).

**Figure 4:**
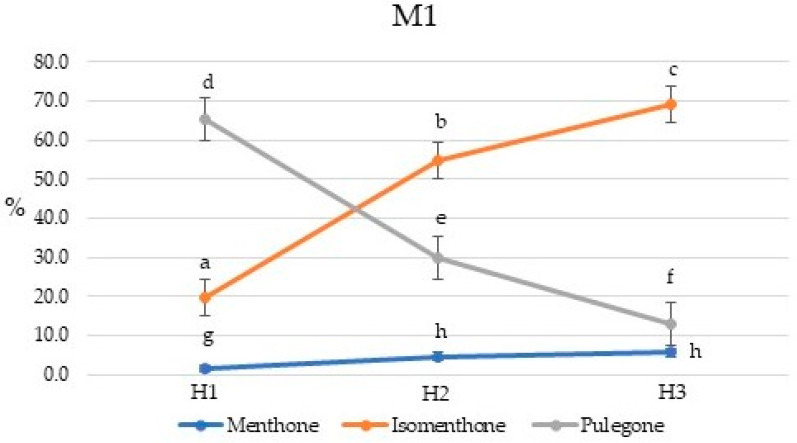
Seasonal variation in the menthone, isomenthone, and pulegone percent concentrations for *Pycnanthemum virginianum* variety M1. Percent concentrations with same letter are not significantly different at *p* ≤ 0.05. H1, H2, and H3 are harvest times, 135, 155, and 170 days after planting, respectively.

**Figure 5:**
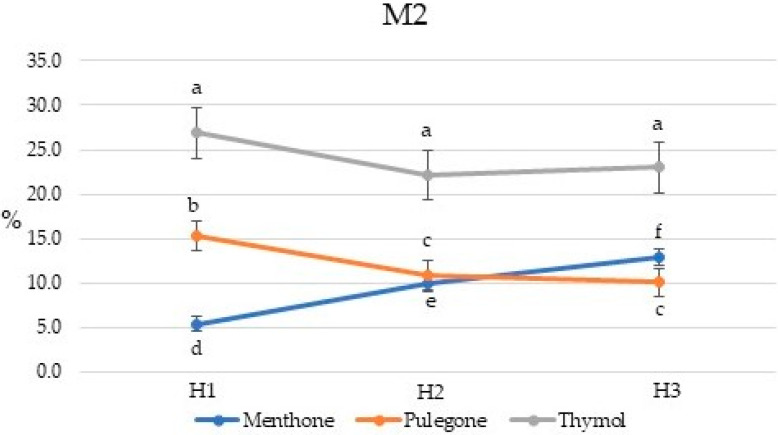
Seasonal variation in the menthone, pulegone, and thymol percent concentrations for *Pycnanthemum virginianum* variety M2. Percent concentrations with same letter are not significantly different at *p* ≤ 0.05. H1, H2, and H3 are harvest times, 135, 155, and 170 days after planting, respectively.

**Figure 6:**
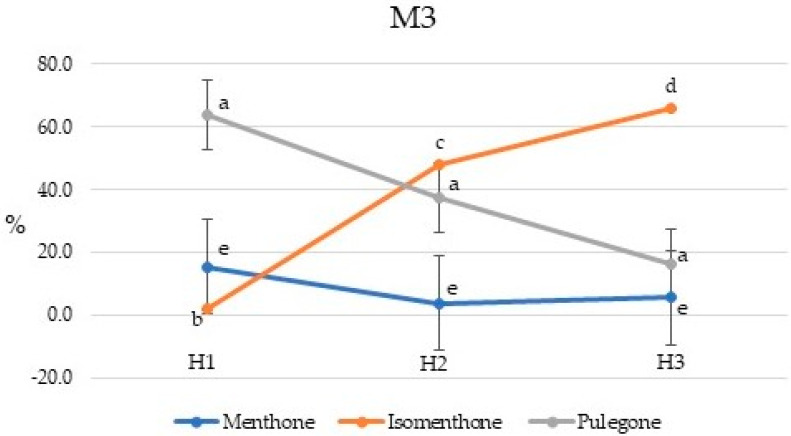
Seasonal variation in the menthone, isomenthone, and pulegone percent concentrations for *Pycnanthemum virginianum* variety M3. Percent concentrations with same letter are not significantly different at *p* ≤ 0.05. H1, H2, and H3 are harvest times, 135, 155, and 170 days after planting, respectively.

**Figure 7:**
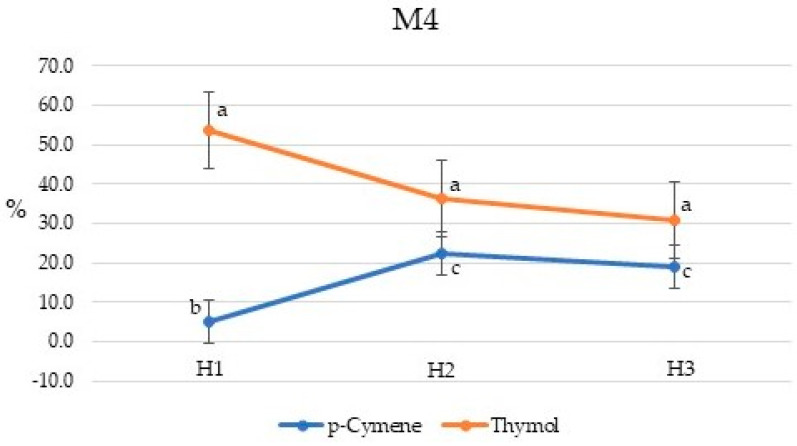
Seasonal variation in the *p*-cymene and thymol percent concentrations for *Pycnanthemum virginianum* variety M4. Percent concentrations with same letter are not significantly different at *p* ≤ 0.05. H1, H2, and H3 are harvest times, 135, 155, and 170 days after planting, respectively.

There are four clearly defined clusters based on the cluster analysis: Cluster 1 can be defined as a pulegone/menthone cluster and comprises five samples, three M1 samples, and two M3 samples, all collected from the first harvest. Cluster 2 is an isomenthone/pulegone cluster and is made up of a variety M1 and M3 samples from harvests two and three. Cluster 3 is a thymol/pulegone cluster made up chiefly of M2 samples. Cluster 4 is a thymol/*p*-cymene chemotype and is made up of variety M4 samples. Chemotype 1, rich in pulegone but also with high concentrations of menthone and isomenthone, is similar in composition to several samples of *Mentha pulegium* L. (pennyroyal) [13] and may, therefore, serve as a substitute herb for pennyroyal. Commercial *M. pulegium* essential oil contains around 84% pulegone (Aromatic Plant Research Center, Lehi, UT, USA). Note that these are early harvest samples of M1 and M3; later-harvested samples of M1 and M3 showed a preponderance of isomenthol with reduced concentrations of pulegone. The concentration of menthone also increased through the season for M1 (Figure 4). Varieties M2 and M3 also showed a seasonal decrease in pulegone and increasing isomenthone (Figure 5 and Figure 6). The decrease in pulegone concentration with a concomitant increase in menthone and isomenthone concentrations is not surprising. Pulegone is the precursor in the biosynthesis of menthone and isomenthone [14,15]. There are several chemotypes of *Thymus vulgaris* L., but one chemotype is rich in thymol and *p*-cymene [16,17], and commercial thyme essential oil (doTERRA International, Pleasant Grove, UT, USA) is composed of 44% thymol and 10% *p*-cymene. Thus, the thymol/*p*-cymene chemotype (cluster 4) of *P. virginianum* could serve as a substitute for thyme. Variety M4 (i.e., cluster 4) showed a significant increase in *p*-cymene concentration coupled with a decrease in thymol concentration (Figure 7).

There is currently very little information on the volatile phytochemistry of other *Pycnanthemum* species. The essential oil composition of *P. incanum*, cultivated in south Alabama, has been determined [18]. The major components in *P. incanum* oil were 1,8-cineole (30.7%), α-terpineol (16.9%), (*E*)-β-caryophyllene (11.0%), borneol (8.2%), and germacrene D (5.0%). *p*-Cymene (0.8%, menthone (0.2%), isomenthone (1.0%), pulegone (1.8%), and thymol (0.3%) were found in relatively low concentrations.

### 2.3. Chemical Composition of Essential Oils

The *P. virginianum* essential oils were analyzed by chiral gas chromatography–mass spectrometry in order to examine the enantiomeric distribution of the terpenoid components. The enantiomeric distributions are compiled in Supplementary Tables S5–S8; a summary of the enantiomeric distributions is shown in Table 2.

There was variability in several of the terpenoid constituents of *P. virginianum* essential oils. α-Thujene showed some variation in enantiomeric distribution between the different varieties; M1 had nearly a racemic mixture, whereas M2 and M4 were predominantly (+)-α-thujene. Camphene was exclusively the (–)-enantiomer in M1, M2, and M4, but it could not be measured in M3. Sabinene showed variation in enantiomeric distribution. M1 and M2 mostly showed the (–)-enantiomer, M3 was exclusively (–)-sabinene, and M4 showed considerable variation through the season. (+)-Linalool was the only enantiomer observed in M2, but the enantiomeric distribution in M4 varied through the season. (–)-α-Terpineol dominated the distribution in M1, M2, and M3, but (+)-α-terpineol was predominant in M4. The (+)-enantiomers were exclusively found for camphene, δ-3-carene, α-terpinene, isomenthone, pulegone, and (*E*)-β-caryophyllene, and the dominant enantiomers for α-phellandrene, *cis*-sabinene hydrate, *trans*-sabinene hydrate, terpinen-4-ol, piperitone, and germacrene D. The (–)-enantiomers were exclusive for β-phellandrene, menthone, and δ-cadinene, and the dominant enantiomer for limonene, δ-elemene, and trans-β-elemene. Consistent with the biosynthesis of monoterpenes in *Mentha* [14,15,19], (+)-pulegone is the apparent precursor of (–)-menthone and (+)-isomenthone in *Pycnanthemum*.

## 3. Materials and Methods

### 3.1. Cultivation of Pycnanthemum virginianum Varieties

The seeds of four varieties of *Pycnanthemum virginianum*, obtained from the USDA germplasm resource, were planted in seed germination flats filled with soilless potting mix and placed in a temperature-controlled greenhouse. The greenhouse was maintained at 26–28 °C/15–18 °C day/night air temperatures with 13 h daylength. The relative humidity (RH) ranged 76–80%. The germinated plants were transplanted into 10-cm pots containing soilless mix and grown in the greenhouse for 1 year.

The propagules with root and rhizome from the 12-month-old potted plants were then transplanted onto raised beds (beds 50 cm wide and 15 cm high) covered with a black plastic sheet with drip tape underneath at the Alabama A&M Winfred Thomas Agricultural Research Station located in Hazel Green, AL (latitude 34°89′ N and longitude 86°56′ W). The soil at the experimental site is a Decatur silty loam (fine, kaolinitic, thermic Rhodic Paleudult). Before making the raised beds, a strip of land was rototilled. A mixture of organic manures, composted chicken manure, and vermicompost to provide an equivalent of 50 kg/ha of nitrogen was incorporated in the soil. Besides organic manure mix, the plants received soluble organic fertilizer at 3-week intervals through the drip irrigation method. The plants were maintained under soil moisture-stress-free conditions. The experimental design was a randomized complete block design with three replications (R). One plant per each variety per replication was harvested at 135, 155, and 170 days after planting (DAP) to assess fresh leaf biomass production and essential oil content and its composition. At each harvest time, the fresh leaves from the plants were collected early morning and placed immediately in a cooler with ice packs for transportation to the laboratory at the University of Alabama in Huntsville for further processing.

### 3.2. Hydrodistillation of Pycnanthemum virginianum

At each harvest, the fresh leaves of *P. virginianum* were chopped and hydrodistilled using a Likens–Nickerson apparatus with continuous extraction with dichloromethane to give the essential oils (Table 3).

### 3.3. Gas Chromatographic Analysis

The *P. virginianum* essential oils were analyzed by gas chromatography with flame ionization detection (GC-FID), gas chromatography–mass spectrometry (GC-MS), and chiral GC-MS as previously described [20]. The percent compositions were determined from raw peak areas from the GC-FID data without standardization. Essential oil components were identified by comparison of the MS fragmentation and retention indices with those in the databases [21,22,23,24]. The enantiomeric distributions were determined from raw peak areas from the chiral GC-MS without standardization.

### 3.4. Statistical Analysis

Each *P. virginianum* variety was analyzed using three replicate plants (when possible) for each harvest time. The data are expressed a means ± standard deviations. Analysis of variance was conducted by one-way ANOVA followed by the Tukey test using Minitab^®^ 18 (Minitab Inc., State College, PA, USA). The interactions between harvesting time and variety were determined using two-way ANOVA. Differences at *p* < 0.05 were considered to be statistically significant. For the agglomerative hierarchical cluster (AHC) analysis, the 31 essential oil compositions were treated as operational taxonomic units (OTUs), and the concentrations (percentages) of 16 of the most abundant essential oil components (1-octen-3-ol, myrcene, *p*-cymene, limonene, γ-terpinene, *cis*-sabinene hydrate, menthone, isomenthone, *trans*-isopulegone, *cis*-piperitenol, pulegone, thymol, carvacrol, unidentified (RI 1345), (*E*)-β-caryophyllene, and germacrene D) were used to determine the chemical associations between the *P. virginianum* essential oil samples using XLSTAT Premium, version 2018.1.1.62926 (Addinsoft, Paris, France). Similarity was determined using Pearson correlation, and clustering was defined using the unweighted pair-group method with arithmetic mean (UPGMA).

## 4. Conclusions

The *Pycnanthemum virginianum* varieties in the study showed significant variation in fresh leaf biomass accumulation and essential oil composition, both between varieties and dramatic seasonal variations. Nevertheless, based on leaf biomass production and chemical profiles, two varieties, M1 and M3, merit further studies to determine yield stability, essential oil yield, composition, and development of cultivation practices for commercial production. For variety M1 at 130 DAP, the biomass yield was good, and the chemical profile was similar to pennyroyal. Variety M2 had a rather unique chemical profile, but the yield was inferior. M4 had a chemical profile similar to thyme, but the biomass yields were low. M3 did not fare well, with half the plants dying, but this may be related to the harvesting plan.

## Figures and Tables

**Table 1:** Major components of *Pycnanthemum virginianum* essential oils cultivated in North Alabama ^a^.

Compound	Percent Composition											
	M1H1 ^b^	M1H2 ^b^	M1H3 ^b^	M2H1 ^b^	M2H2 ^b^	M2H3 ^b^	M3H1 ^c^	M3H2 ^d^	M3H3 ^d^	M4H1 ^b^	M4H2 ^b^	M4H3 ^b^
1-Octen-3-ol	2.0 ± 0.2	2.0 ± 0.2	1.5 ± 0.2	2.6 ± 0.1	2.0 ± 0.4	2.2 ± 0.7	3.5 ± 0.4	1.8	1.9	3.2 ± 0.5	5.5 ± 0.7	5.8 ± 1.5
Myrcene	0.4 ± 0.1	0.4 ± 0.0	0.4 ± 0.0	3.1 ± 1.1	2.6 ± 0.9	1.9 ± 0.7	0.1 ± 0.1	0.4	0.4	1.3 ± 1.0	0.8 ± 0.5	0.5 ± 0.1
*p*-Cymene	0.1 ± 0.0	tr	tr	7.1 ± 0.8	8.7 ± 2.1	7.4 ± 0.7	0.2 ± 0.1	tr	0.3	5.0 ± 2.7	22.5 ± 2.4	18.9 ± 8.8
Limonene	1.9 ± 0.2	1.3 ± 0.1	1.1 ± 0.2	5.8 ± 1.2	5.5 ± 1.4	4.1 ± 1.0	1.0 ± 0.7	1.5	1.2	0.7 ± 0.3	0.6 ± 0.2	0.5 ± 0.1
γ-Terpinene	tr	tr	tr	2.4 ± 0.3	1.7 ± 0.6	0.9 ± 0.2	nd	nd	tr	3.9 ± 2.4	1.7 ± 0.5	1.2 ± 0.3
*cis*-Sabinene hydrate	tr	tr	tr	0.6 ± 0.1	0.6 ± 0.1	0.7 ± 0.1	tr	tr	tr	1.0 ± 0.2	2.5 ± 0.8	2.7 ± 1.0
Menthone	1.5 ± 0.6	4.7 ± 0.6	5.6 ± 0.9	5.4 ± 0.9	9.9 ± 0.1	12.9 ± 1.2	15.3 ± 15.1	3.8	5.6	0.3 ± 0.3	0.1 ± 0.0	0.9 ± 0.7
Isomenthone	19.9 ± 6.9	54.7 ± 3.8	69.3 ± 1.4	0.1 ± 0.0	0.4 ± 0.4	1.8 ± 1.3	1.8 ± 0.1	48.0	65.8	2.7 ± 3.2	0.4 ± 0.6	6.3 ± 5.9
*trans*-Isopulegone	1.6 ± 0.1	1.1 ± 0.1	0.9 ± 0.0	3.6 ± 0.2	4.1 ± 0.4	4.1 ± 0.8	1.6 ± 0.2	1.2	1.1	0.2 ± 0.4	nd	0.2 ± 0.3
*cis*-Piperitenol	nd	nd	nd	2.6 ± 0.2	2.3 ± 0.3	2.2 ± 0.4	nd	nd	0.1	tr	nd	0.1 ± 0.1
Pulegone	65.3 ± 8.4	29.8 ± 3.9	13.0 ± 1.8	15.3 ± 1.8	10.8 ± 1.4	10.1 ± 1.6	63.8 ± 11.2	37.3	16.1	11.8 ± 15.4	0.6 ± 0.9	2.3 ± 1.8
Thymol	0.4 ± 0.1	0.2 ± 0.2	tr	26.9 ± 4.1	22.1 ± 2.3	23.0 ± 1.3	0.3 ± 0.4	tr	0.6	53.8 ± 15.2	36.5 ± 5.4	30.9 ± 5.3
Carvacrol	tr	tr	tr	1.5 ± 0.2	1.4 ± 0.1	1.4 ± 0.1	tr	nd	tr	1.3 ± 0.4	1.1 ± 0.3	0.7 ± 0.1
Unidentified (RI 1345)	0.1 ± 0.0	0.1 ± 0.0	tr	7.5 ± 0.7	10.7 ± 0.8	10.7 ± 2.4	0.1 ± 0.1	nd	0.3	0.2 ± 0.2	nd	0.6 ± 0.6
(*E*)-β-Caryophyllene	1.2 ± 0.2	0.6 ± 0.5	1.3 ± 0.2	1.1 ± 0.1	1.2 ± 0.2	1.2 ± 0.2	0.8 ± 1.1	1.0	1.1	1.8 ± 0.2	1.7 ± 0.4	1.6 ± 0.7
Germacrene D	1.2 ± 0.4	0.7 ± 0.5	1.1 ± 0.0	1.8 ± 0.1	1.5 ± 0.2	1.4 ± 0.1	0.2 ± 0.3	0.7	1.0	1.4 ± 0.5	0.8 ± 0.0	1.0 ± 0.6

^a^ M1 = Variety #1, M2 = Variety #2, M3 = Variety #3, M4 = Variety #4. H1 = Harvest #1 (30 September 2020, 135 days after transplanting), H2 = Harvest #2 (19 October 2020, 155 days after transplanting), H3 = Harvest #3 (4 November 2020, 170 days after transplanting). tr = trace (< 0.05%), nd = not detected. ^b^ Average of three different plants ± standard deviations. ^c^ Average of two plants (third plant died) ± standard deviations. ^d^ Only one plant survived.

**Table 2:** Enantiomeric distribution of terpenoid constituents of *Pycnanthemum virginianum* ^a^.

Compound	M1	M2	M3	M4
α-Thujene	54:46	72:28	---	76:24
α-Pinene	24:76	58:42	23:77	72:28
Camphene	100:0	100:0	---	100:0
Sabinene	30:70	0:100	28:72	variable ^b^
β-Pinene	46:54	54:46	46:54	31:69
α-Phellandrene	---	95:5	---	96:4
δ-3-Carene	---	100:0	---	100:0
α-Terpinene	---	100:0	---	100:0
Limonene	6:94	0:100	7:93	variable ^c^
β-Phellandrene	---	0:100	---	0:100
*cis*-Sabinene hydrate	---	95:5	---	99:1
Linalool	---	100:0	---	variable ^d^
*trans*-Sabinene hydrate	---	78:22	---	82:18
Menthone	0:100	0:100	0:100	0:100
Isomenthone	100:0	100:0	100:0	100:0
Borneol	---	0:100	---	0:100
Terpinen-4-ol	---	65:35	---	66:34
α-Terpineol	9:91	32:68	10:90	variable ^e^
Pulegone	100:0	100:0	100:0	100:0
Piperitone	94:6	---	89:11	---
δ-Elemene	---	---	---	variable ^f^
α-Copaene	---	---	---	100:0
*trans*-β-Elemene	15:85	20:80	16:84	6:94
(*E*)-β-Caryophyllene	100:0	100:0	100:0	100:0
Germacrene D	91:9	94:6	91:9	80:20
δ-Cadinene	0:100	0:100	0:100	0:100

^a^ Average enantiomeric distributions, % (+)-enantiomer : % (–)-enantiomer, for each variety. ^b^ (+)-enantiomer ranged 0–72%. ^c^ Mostly (–)-enantiomer, but one sample with 49% (+)-limonene. ^d^ (+)-enantiomer ranged 19–100%. ^e^ Mostly (+)-enantiomer, but one sample with only 33% (+)-linalool. ^f^ (+)-enantiomer ranged 0–50%.

**Table 3:** Hydrodistillation details of *Pycnanthemum virginianum* cultivated in North Alabama.

Sample ^a^	Plant Mass (g)	Essential Oil Yield (mg)	% Yield	Essential Oil Color
M1H1R1	104.08	1127.6	1.083	pale yellow
M1H1R2	105.70	1164.6	1.102	pale yellow
M1H1R3	122.45	1548.9	1.265	pale yellow
M2H1R1	82.71	704.9	0.852	yellow
M2H1R2	70.22	580.0	0.826	yellow
M2H1R3	75.80	791.6	1.044	yellow
M3H1R1	40.13	302.3	0.753	pale yellow
M3H1R2	0.00	plant died	---	---
M3H1R3	74.15	572.1	0.772	pale yellow
M4H1R1	30.67	301.2	0.982	yellow
M4H1R2	35.31	345.8	0.979	yellow
M4H1R3	73.80	825.5	1.119	orange
M1H2R1	139.76	1330.7	0.952	pale yellow
M1H2R2	133.69	1093.3	0.818	pale yellow
M1H2R3	167.15	1862.5	1.114	pale yellow
M2H2R1	74.47	596.6	0.801	yellow
M2H2R2	59.86	463.1	0.774	yellow
M2H2R3	132.61	1003.9	0.757	yellow
M3H2R1	182.68	2157.9	1.181	pale yellow
M3H2R2	0.00	plant died	---	---
M3H2R3	0.00	plant died	---	---
M4H2R1	8.10	92.6	1.143	orange
M4H2R2	60.66	252.3	0.416	orange
M4H2R3	32.81	208.2	0.635	orange
M1H3R1	100.14	582.0	0.581	pale yellow
M1H3R2	110.22	897.3	0.814	pale yellow
M1H3R3	59.71	556.9	0.933	pale yellow
M2H3R1	39.93	298.4	0.747	yellow
M2H3R2	53.23	510.8	0.960	yellow
M2H3R3	70.02	495.8	0.708	yellow
M3H3R1	0.00	plant died	---	---
M3H3R2	0.00	plant died	---	---
M3H3R3	110.07	945.5	0.859	yellow
M4H3R1	53.98	320.2	0.593	orange
M4H3R2	29.91	108.9	0.364	orange
M4H3R3	19.84	143.9	0.725	orange

^a^ M indicates the *P. virginianum* variety; H is the harvest (H1 = 135 days after planting (DAP), H2 = 155 DAP, and H3 = 170 DAP); R is the number of replicates of each plant variety (three replicates of each variety).

## Data Availability

All of the data are available within this manuscript and the Supplementary Tables.

## Supplementary Materials

The following are available online at https://www.mdpi.com/article/10.3390/plants10071397/s1, Figure S1: Huntsville, Alabama, weather data, 2020. Table S1: Essential oil composition of *Pycnanthemum virginianum* variety M1, Table S2: Essential oil composition of *Pycnanthemum virginianum* variety M2, Table S3: Essential oil composition of *Pycnanthemum virginianum* variety M3, Table S4: Essential oil composition of *Pycnanthemum virginianum* variety M4, Table S5: Enantiomeric distribution of terpenoid constituents of *Pycnanthemum virginianum*, variety M1, Table S6: Enantiomeric distribution of terpenoid constituents of *Pycnanthemum virginianum*, variety M2, Table S7: Enantiomeric distribution of terpenoid constituents of *Pycnanthemum virginianum*, variety M3, Table S8: Enantiomeric distribution of terpenoid constituents of *Pycnanthemum virginianum*, variety M4.
