# Supplementary material for: Essential Oils of Four Virginia Mountain Mint (Pycnanthemum virginianum) Varieties Grown in North Alabama
Source: Plants (Basel). 2021 Jul 8;10(7):1397. doi: 10.3390/plants10071397 (PMC8309247; doi:10.3390/plants10071397)
Supplement: Supplementary file 1 [file plants-10-01397-s001.zip › plants-1268847 Supplementary Tables.pdf]

Supplementary Table S1. Essential oil composition of *Pycnanthemum virginianum* variety M1.

RI(calc)	RI(db)	Compound	M1-R1-H1	M1-R2-H1	M1-R3-H1	M1-R1-H2	M1-R2-H2	M1-R3-H2	M1-R1-H3	M1-R2-H3	M1-R3-H3
925	925	α-Thujene	tr	tr	tr	tr	tr	tr	tr	tr	tr
932	932	α-Pinene	0.2	0.1	0.3	0.2	0.1	0.2	0.2	0.2	0.2
949	950	Camphene	tr	tr	tr	tr	tr	tr	tr	tr	tr
951	951	3-Methylcyclohexanone	tr	tr	tr	tr	tr	tr	tr	tr	tr
952	953	Thuja-2,4(10)-diene	---	---	---	tr	---	---	tr	---	---
971	971	Sabinene	0.1	0.1	0.1	0.1	0.1	0.1	0.1	0.1	0.1
978	978	β-Pinene	0.3	0.2	0.3	0.3	0.2	0.3	0.2	0.2	0.3
979	978	1-Octen-3-ol	1.8	2.2	1.9	2.0	2.1	1.8	1.7	1.4	1.5
984	983	3-Octanone	0.5	0.3	0.5	0.5	0.5	0.6	0.5	0.5	0.5
989	989	Myrcene	0.5	0.3	0.5	0.4	0.4	0.4	0.3	0.4	0.3
997	996	3-Octanol	0.1	0.1	0.1	0.1	0.1	0.1	0.1	0.1	0.1
1004	1004	*p*-Mentha-1(7),8-diene	tr	tr	tr	tr	tr	tr	tr	tr	tr
1017	1017	α-Terpinene	tr	tr	tr	tr	tr	tr	tr	tr	tr
1024	1024	*p*-Cymene	0.1	0.1	0.1	0.1	tr	tr	tr	0.1	tr
1029	1030	Limonene	1.8	1.7	2.1	1.4	1.2	1.4	1.1	1.4	1.0
1031	1029	β-Phellandrene	tr	tr	tr	---	tr	---	---	tr	---
1032	1030	1,8-Cineole	0.3	0.2	0.3	0.3	0.3	0.3	0.3	0.3	0.3
1035	1034	(*Z*)-β-Ocimene	tr	tr	tr	tr	tr	tr	tr	tr	tr
1045	1045	(*E*)-β-Ocimene	0.2	0.1	0.1	0.1	0.1	0.1	0.2	0.2	0.1
1057	1057	γ-Terpinene	tr	tr	tr	tr	tr	tr	tr	tr	tr
1070	1069	*cis*-Sabinene hydrate	tr	tr	tr	tr	tr	tr	tr	tr	tr
1083	1079	1-Nonen-3-ol	tr	tr	tr	tr	tr	tr	tr	tr	tr
1085	1086	Terpinolene	tr	tr	tr	tr	tr	tr	tr	tr	tr
1090	1091	*p*-Cymenene	tr	tr	tr	tr	tr	tr	tr	tr	tr
1100	1099	Linalool	tr	tr	tr	0.1	tr	tr	tr	tr	tr
1105	1107	Nonanal	tr	tr	tr	tr	tr	tr	tr	tr	tr
1107	1107	1-Octen-3-yl acetate	tr	tr	tr	tr	tr	tr	tr	tr	tr
1145	1140	*trans*-Sabinol	---	---	---	---	---	tr	0.1	tr	tr
1146	1141	*cis*-Verbenol	---	---	---	---	---	---	---	tr	---

1146	1144	*trans*-Tagetone	---	---	---	---	---	tr	---	---	tr
1150	1149	*p*-Menth-3-en-8-ol	0.1	0.1	tr	---	---	---	---	---	---
1150	1145	*trans*-Verbenol	0.1	0.1	0.1	0.1	0.1	0.1	0.1	0.1	0.1
**1156**	**1156**	**Menthone**	**2.2**	**0.9**	**1.5**	**4.2**	**5.4**	**4.4**	**5.8**	**6.4**	**4.7**
**1169**	**1166**	**Isomenthone**	**26.7**	**12.9**	**20.2**	**50.5**	**58.1**	**55.4**	**70.5**	**69.6**	**67.8**
1175	1171	*p*-Mentha-1,5-dien-8-ol	---	---	---	---	---	---	---	tr	---
1176	1173	Borneol	---	---	---	tr	---	tr	tr	tr	tr
1178	1176	*trans*-Isopulegone	1.5	1.7	1.6	1.2	1.1	1.2	0.9	0.9	0.9
1182	1180	Terpinen-4-ol	tr	tr	tr	tr	tr	tr	tr	0.1	tr
1184	1184	1-Decen-3-ol	tr	tr	tr	tr	---	---	---	---	---
1187	1184	6-Methyl-2-vinyl-5-hepten-1-ol	tr	tr	tr	tr	tr	---	tr	0.1	---
1193	1190	*neoiso*-Menthol	---	---	---	---	---	---	---	tr	---
1196	1195	α-Terpineol	0.3	0.3	0.3	0.3	0.3	0.3	0.3	0.3	0.2
1201	1201	*cis*-Piperitenol	---	---	---	---	---	---	---	tr	---
1207	1208	Decanal	---	---	---	tr	tr	tr	tr	0.1	tr
1208	1208	Verbenone	---	---	---	0.1	0.1	0.1	0.1	0.1	0.1
1218	1217	3-Isopropylbenzaldehyde	---	---	---	0.1	0.1	0.1	0.2	0.3	0.3
1226	1230	Cuminaldehyde	0.2	0.1	0.1	0.2	0.1	0.1	---	---	---
**1243**	**1241**	**Pulegone**	**57.3**	**74.0**	**64.7**	**33.4**	**25.7**	**30.1**	**12.5**	**11.4**	**15.0**
1254	1254	Piperitone	0.1	tr	0.1	0.2	0.3	0.2	0.9	1.0	1.0
1258	1261	Pulegone oxide A	---	tr	tr	---	---	---	---	---	---
1269	1270	*iso*-Piperitenone	tr	0.1	0.1	0.1	0.1	tr	tr	tr	tr
1287	1287	Pulegone oxide B	0.1	0.3	0.1	---	---	0.1	---	0.2	0.2
1289	1289	Thymol	0.5	0.4	0.4	0.3	0.1	tr	tr	0.1	tr
1297	1296	Carvacrol	tr	tr	tr	tr	tr	---	---	tr	---
1297	1300	Pulegone oxide C	---	tr	---	---	---	---	---	---	---
1308	1309	4-Vinylguaicol	tr	---	tr	tr	---	---	tr	tr	tr
1331	1326	Bicycloelemene	tr	tr	tr	tr	tr	---	tr	tr	tr
1335	1335	δ-Elemene	0.3	tr	0.3	0.2	tr	tr	0.2	0.2	0.2
1337	1339	Piperitenone	0.3	0.3	0.3	0.2	0.1	0.1	0.1	0.1	0.2
1338	---	Unidentified	0.1	tr	0.1	0.1	0.1	---	tr	tr	---

1342	1348	Mint furanone isomer A	---	tr	---	---	---	---	---	---	---
1344	1349	Mint furanone isomer B	---	tr	tr	---	---	---	---	---	---
1375	1375	α-Copaene	tr	tr	tr	tr	tr	tr	tr	tr	tr
1381	1383	*cis*-β-Elemene	tr	tr	tr	tr	tr	tr	tr	tr	tr
1383	1382	β-Bourbonene	0.1	0.1	0.1	0.1	tr	0.1	0.2	0.3	0.3
1385	1385	α-Bourbonene	tr	tr	tr	tr	tr	tr	tr	tr	tr
1389	1390	*trans*-β-Elemene	0.3	0.2	0.3	0.2	0.1	0.2	0.2	0.3	0.3
1391	1392	(*Z*)-Jasmone	0.1	0.1	0.1	0.1	0.2	0.1	0.1	0.1	0.1
1419	1417	(*E*)-β-Caryophyllene	1.4	1.0	1.2	1.0	0.1	0.9	1.1	1.4	1.4
1429	1430	β-Copaene	tr	tr	tr	tr	1.0	tr	tr	tr	0.1
1432	1432	*trans*-α-Bergamotene	0.2	0.2	0.2	0.1	tr	0.1	0.1	0.1	0.2
1439	1439	(*Z*)-β-Farnesene	---	---	---	---	---	---	---	---	tr
1440	1443	Citronellyl propionate	---	---	---	---	---	tr	---	---	---
1443	1447	*iso*-Germacrene D	---	---	---	---	---	tr	tr	tr	tr
1452	1452	(*E*)-β-Farnesene	---	---	---	---	---	tr	---	---	tr
1454	1453	α-Humulene	0.2	0.2	0.2	0.1	0.1	0.1	0.2	0.2	0.2
1467	1465	*cis*-Muurola-4(14),5-diene	---	---	---	---	---	tr	---	tr	tr
1474	1478	γ-Muurolene	---	---	---	---	---	tr	---	tr	tr
1480	1480	Germacrene D	1.5	0.8	1.4	1.1	0.2	0.7	1.1	1.1	1.2
1483	1483	*trans*-β-Bergamotene	tr	tr	tr	tr	0.9	tr	tr	tr	0.1
1484	1485	γ-Thujaplicin	---	tr	---	---	---	---	---	---	---
1489	1489	(*Z*,*E*)-α-Farnesene	---	---	---	tr	---	tr	tr	tr	tr
1494	1497	Bicyclogermacrene	0.1	0.1	0.1	0.1	tr	0.1	0.1	0.1	0.1
1503	1504	(*E*,*E*)-α-Farnesene	0.3	0.2	0.3	0.2	0.1	0.1	0.2	0.2	0.3
1506	1508	β-Bisabolene	tr	tr	tr	tr	0.2	tr	tr	tr	tr
1512	1512	γ-Cadinene	tr	tr	tr	tr	tr	tr	tr	tr	tr
1517	1518	δ-Cadinene	tr	tr	tr	tr	tr	tr	tr	tr	tr
1523	1523	β-Sesquiphellandrene	0.2	0.1	0.2	0.1	tr	0.1	0.1	0.1	0.1
1547	1546	α-Elemol	tr	tr	tr	tr	0.1	tr	tr	tr	tr
1557	1557	Germacrene B	tr	tr	tr	tr	tr	tr	tr	tr	tr
1560	1560	(*E*)-Nerolidol	tr	tr	tr	tr	tr	tr	tr	tr	tr

1575	1576	Spathulenol	tr	tr	tr	tr	tr	tr	tr	tr	tr
1580	1577	Caryophyllene oxide	0.1	0.1	tr	tr	tr	0.1	0.1	0.1	0.2
1607	1607	Humulene epoxide I	---	tr	tr	---	0.1	tr	tr	tr	tr
1627	1629	*iso*-Spathulenol	tr	0.1	tr	tr	tr	0.1	tr	tr	tr
1631	1630	Caryophylla-4(12),8(13)-dien-5α-ol	---	---	---	---	---	---	---	tr	---
1635	1636	Caryophylla-4(12),8(13)-dien-5β-ol	---	---	---	---	---	---	tr	tr	---
1637	1639	*cis*-Guaia-3,9-dien-11-ol	tr	tr	tr	tr	0.1	---	tr	tr	tr
1638	1644	*allo*-Aromadendrene epoxide	---	---	---	---	tr	tr	---	---	---
1640	1638	τ-Cadinol	tr	tr	tr	tr	---	tr	tr	tr	tr
1642	1640	τ-Muurolol	tr	tr	tr	tr	---	tr	tr	tr	tr
1654	1652	α-Cadinol	tr	tr	tr	tr	tr	tr	tr	tr	tr
1656	1658	Selin-11-en-4α-ol	---	---	---	---	---	---	tr	tr	---
1682	1683	Germacra-4(15),5,10(14)-trien-1α-ol	---	---	---	---	---	---	---	tr	---

Supplementary Table S2. Essential oil composition of *Pycnanthemum virginianum* variety M2.

RI(calc)	RI(db)	Compound	M2-R1-H1	M2-R2-H1	M2-R3-H1	M2-R1-H2	M2-R2-H2	M2-R3-H2	M2-R1-H3	M2-R2-H3	M2-R3-H3
925	925	α-Thujene	0.4	0.3	1.4	0.8	0.1	0.7	tr	0.3	0.2
932	932	α-Pinene	0.2	0.1	0.7	0.4	0.1	0.3	tr	0.2	0.1
949	950	Camphene	0.1	tr	0.2	0.1	tr	0.1	tr	0.1	tr
952	951	3-Methylcyclohexanone	tr	tr	tr	---	tr	tr	tr	tr	tr
953	953	Thuja-2,4(10)-diene	---	---	---	---	---	tr	---	---	---
961	960	Benzaldehyde	---	---	---	---	tr	tr	tr	tr	tr
972	971	Sabinene	0.2	0.1	0.4	0.2	0.1	0.3	tr	0.2	0.2
977	978	β-Pinene	0.1	0.1	0.3	0.2	0.1	0.2	tr	0.1	0.1
980	978	1-Octen-3-ol	2.6	2.6	2.7	1.6	2.1	2.3	1.4	2.4	2.7
984	983	3-Octanone	0.4	0.3	0.5	0.4	0.4	0.5	0.3	0.4	0.5
990	989	Myrcene	2.9	2.2	4.4	3.0	1.6	3.3	1.1	2.3	2.4
997	996	3-Octanol	0.2	0.2	0.2	0.2	0.2	0.2	0.2	0.2	0.2
1005	1004	*p*-Mentha-1(7),8-diene	tr	tr	tr	tr	tr	tr	tr	tr	tr
1007	1006	α-Phellandrene	0.1	0.1	0.2	0.1	tr	0.1	tr	tr	tr
1009	1008	δ-3-Carene	tr	tr	0.1	0.1	tr	tr	tr	tr	tr
1017	1017	α-Terpinene	1.2	0.9	1.5	0.8	0.5	1.0	0.2	0.5	0.5
1020	1022	*m*-Cymene	tr	tr	tr	tr	tr	tr	tr	tr	tr
**1026**	**1024**	***p*-Cymene**	**6.8**	**6.5**	**8.0**	**10.9**	**6.7**	**8.6**	**6.8**	**8.2**	**7.1**
1028	1028	2-Acetyl-3-methylfuran	---	---	---	tr	---	tr	0.1	tr	tr
**1030**	**1030**	**Limonene**	**5.5**	**4.6**	**7.1**	**6.5**	**3.9**	**6.1**	**2.9**	**4.8**	**4.5**
1031	1029	β-Phellandrene	0.1	0.1	0.2	---	0.1	---	---	---	---
1032	1030	1,8-Cineole	0.9	0.8	1.0	0.8	0.8	1.1	0.7	1.0	1.2
1033	1033	Benzyl alcohol	---	---	---	---	---	---	tr	---	---
1035	1034	(*Z*)-β-Ocimene	0.1	0.1	0.2	0.1	0.1	0.2	0.1	0.1	0.1
1046	1045	(*E*)-β-Ocimene	1.2	0.9	1.5	1.0	0.7	1.4	0.4	0.5	0.7
1058	1057	γ-Terpinene	2.5	2.1	2.7	1.7	1.1	2.3	0.7	1.0	1.2
1069	1069	*cis*-Sabinene hydrate	0.6	0.7	0.6	0.6	0.7	0.5	0.9	0.7	0.6
1080	1079	1-Nonen-3-ol	tr	tr	tr	tr	tr	tr	tr	tr	tr
1085	1086	Terpinolene	0.1	0.1	0.1	0.1	0.1	0.1	tr	tr	0.1

1090	1091	*p*-Cymenene	tr	tr	tr	0.1	tr	tr	0.1	tr	tr
1100	1099	Linalool	0.2	0.2	0.2	0.1	0.2	0.2	0.2	0.2	0.2
1101	1099	*trans*-Sabinene hydrate	0.1	0.1	0.1	0.2	0.2	0.1	0.3	0.2	0.2
1105	1105	Nonanal	---	---	tr	---	tr	tr	tr	0.1	tr
1107	1107	1-Octen-3-yl acetate	tr	tr	tr	tr	tr	tr	0.1	0.1	0.1
1112	1112	2,4-Dimethylhepta-2,4-dienal	---	---	---	---	---	---	tr	---	---
1117	1112	2,4-Dimethylhepta-2,4-dienal	---	---	---	---	---	---	tr	---	---
1122	1120	*trans*-*p*-Mentha-2,8-dien-1-ol	0.2	0.2	0.2	0.3	0.3	0.3	0.3	0.2	0.2
1124	1124	*cis*-*p*-Menth-2-en-1-ol	tr	tr	tr	tr	tr	tr	tr	tr	tr
1132	1132	*cis*-Limonene oxide	---	---	---	---	---	---	tr	tr	---
1137	1137	*cis*-*p*-Mentha-2,8-dien-1-ol	0.2	0.2	0.2	0.3	0.3	0.3	0.3	0.2	0.2
1140	1140	*trans*-Sabinol	---	---	---	tr	tr	tr	tr	tr	tr
1142	1142	*trans*-*p*-Menth-2-en-1-ol	---	---	---	---	tr	---	tr	---	---
1146	1145	*trans*-Verbenol	tr	tr	tr	tr	0.1	tr	0.1	tr	tr
1151	1150	*p*-Menth-3-en-8-ol	0.1	tr	tr	tr	tr	tr	tr	tr	tr
**1157**	**1156**	**Menthone**	**4.7**	**4.9**	**6.5**	**9.9**	**10.0**	**9.8**	**11.7**	**12.8**	**14.2**
1160	1160	*iso*-Isopulegol	---	---	---	tr	tr	tr	tr	tr	tr
1163	1164	Menthofuran	---	---	---	---	tr	tr	tr	tr	tr
1166	1166	Isomenthone	0.2	0.1	0.1	0.1	0.8	0.2	1.3	0.8	3.2
1170	1170	Umbellulone	---	---	---	---	0.1	tr	tr	---	---
1173	1173	Borneol	tr	tr	tr	tr	0.1	0.1	0.1	tr	tr
**1177**	**1176**	***trans*-Isopulegone**	**3.7**	**3.7**	**3.4**	**4.5**	**4.2**	**3.7**	**5.0**	**3.8**	**3.6**
1181	1180	Terpinen-4-ol	0.5	0.5	0.5	0.5	0.6	0.6	0.5	0.4	0.5
1188	1188	*p*-Cymen-8-ol	---	---	---	0.1	0.1	0.1	0.1	0.1	tr
1195	1195	α-Terpineol	0.4	0.4	0.4	0.3	0.4	0.4	0.3	0.4	0.4
1201	1201	*cis*-Piperitenol	2.7	2.8	2.4	2.3	2.7	2.1	2.7	2.1	2.0
1206	1206	Decanal	---	---	---	tr	---	---	tr	---	---
1208	1208	Verbenone	tr	0.1	0.1	0.1	0.1	tr	0.1	tr	tr
1225	1224	Thymol methyl ether	0.3	0.4	0.3	0.5	0.6	0.5	0.6	1.1	0.9
**1241**	**1241**	**Pulegone**	**16.6**	**16.0**	**13.3**	**9.7**	**12.5**	**10.3**	**11.9**	**9.0**	**9.3**
1264	1267	Piperitone	---	---	---	---	---	---	0.2	0.1	0.1

1269	1270	*iso*-Piperitenone	---	---	---	---	---	---	0.2	---	---
1287	1287	Pulegone oxide B	0.1	0.2	0.1	---	---	---	---	0.3	---
**1292**	**1289**	**Thymol**	**26.8**	**31.0**	**22.8**	**20.0**	**24.5**	**21.9**	**23.8**	**23.8**	**21.5**
1298	1291	*p*-Cymen-7-ol	---	---	---	---	0.1	---	---	---	---
1300	1296	Carvacrol	1.5	1.7	1.4	1.3	1.6	1.4	1.5	1.5	1.4
1332	1326	Bicycloelemene	tr	tr	tr	tr	tr	tr	tr	tr	tr
1335	1335	δ-Elemene	0.2	0.1	0.2	0.1	0.1	0.1	0.1	0.1	0.1
**1345**	**---**	**Unidentified**	**7.6**	**8.2**	**6.8**	**11.6**	**10.5**	**10.0**	**13.5**	**9.7**	**9.0**
1347	1348	α-Cubebene	---	---	---	---	---	---	---	---	tr
1350	---	Unidentified	0.7	0.8	0.9	1.7	2.2	2.1	2.1	4.2	4.7
1353	1356	Eugenol	---	---	---	---	---	---	---	tr	tr
1360	1358	Piperitenone oxide	tr	tr	tr	tr	tr	tr	tr	tr	tr
1375	1375	α-Copaene	tr	tr	tr	tr	0.1	tr	tr	tr	tr
1382	1383	*cis*-β-Elemene	tr	tr	tr	tr	tr	tr	tr	tr	tr
1383	1382	β-Bourbonene	0.1	0.1	0.1	0.1	0.1	0.1	0.2	0.2	0.2
1386	1385	α-Bourbonene	---	---	---	---	---	---	---	tr	tr
1387	1387	β-Cubebene	---	---	---	---	---	tr	---	tr	tr
1389	1390	*trans*-β-Elemene	0.3	0.2	0.3	0.2	0.3	0.2	0.2	0.2	0.2
1392	1392	(*Z*)-Jasmone	0.1	tr	0.1	tr	0.1	0.1	0.1	tr	0.1
**1419**	**1417**	**(*E*)-β-Caryophyllene**	**1.2**	**1.0**	**1.2**	**1.2**	**1.4**	**1.1**	**1.5**	**1.1**	**1.1**
1429	1430	β-Copaene	0.1	0.1	0.1	0.1	0.1	0.1	0.1	0.1	0.1
1432	1432	*trans*-α-Bergamotene	0.3	0.3	0.3	0.2	0.4	0.3	0.3	0.3	0.3
1436	---	Unidentified	0.7	0.5	0.9	0.9	1.1	1.0	0.6	0.6	0.4
1440	1440	(*Z*)-β-Farnesene	---	---	---	---	---	---	---	tr	tr
1443	1447	*iso*-Germacrene D	---	---	---	---	---	---	---	tr	tr
1447	1448	(2*E*,6*Z*)-Dodecadienal	---	---	---	---	---	---	---	tr	tr
1452	1451	(*E*)-β-Farnesene	0.1	tr	0.1	tr	0.1	tr	tr	tr	tr
1454	1453	α-Humulene	0.1	0.1	0.1	0.1	0.2	0.1	0.2	0.1	0.1
1458	1458	*allo*-Aromadendrene	---	---	---	---	tr	---	tr	tr	tr
1474	1478	γ-Muurolene	tr	tr	tr	tr	0.1	tr	tr	tr	tr
1476	1475	γ-Gurjunene	tr	tr	tr	tr	tr	tr	tr	tr	tr

1480	1480	Germacrene D	1.9	1.8	1.8	1.2	1.7	1.5	1.3	1.5	1.4
1483	1483	*trans*-β-Bergamotene	0.1	0.1	0.1	0.1	0.1	0.1	0.1	0.1	0.1
1486	1488	δ-Selinene	---	---	---	---	---	---	tr	---	---
1487	1487	β-Selinene	tr	tr	tr	tr	tr	tr	tr	tr	tr
1490	1491	Viridiflorene	tr	tr	tr	tr	tr	tr	tr	tr	tr
1490	1490	γ-Amorphene	tr	tr	tr	tr	tr	tr	tr	tr	tr
1494	1497	Bicyclogermacrene	0.3	0.3	0.3	0.2	0.3	0.3	0.2	0.2	0.2
1497	1497	α-Muurolene	tr	tr	tr	tr	tr	tr	tr	tr	tr
1503	1504	(*E*,*E*)-α-Farnesene	0.4	0.3	0.4	0.3	0.3	0.4	0.2	0.2	0.3
1507	1508	β-Bisabolene	tr	tr	tr	tr	tr	tr	tr	tr	tr
1512	1512	γ-Cadinene	0.1	tr	tr	tr	tr	tr	tr	tr	tr
1517	1518	δ-Cadinene	0.1	0.1	0.1	0.1	0.1	0.1	0.1	0.1	0.1
1523	1523	β-Sesquiphellandrene	0.3	0.2	0.2	0.2	0.3	0.2	0.2	0.3	0.2
1547	1546	α-Elemol	tr	tr	tr	tr	tr	tr	tr	tr	tr
1557	1557	Germacrene B	tr	tr	tr	tr	tr	tr	tr	tr	tr
1560	1560	(*E*)-Nerolidol	tr	tr	tr	tr	tr	tr	tr	tr	tr
1575	1576	Spathulenol	0.1	0.2	0.1	0.2	0.3	0.2	0.3	0.3	0.3
1580	1577	Caryophyllene oxide	0.1	0.1	0.1	0.3	0.3	0.2	0.5	0.2	0.2
1608	1607	Humulene epoxide I	---	---	---	tr	---	---	0.1	---	tr
1627	1629	*iso*-Spathulenol	0.1	0.1	0.1	0.1	0.1	0.1	0.1	0.1	0.1
1635	1635	Caryophylla-4(12),8(13)-dien-5β-ol	---	---	---	---	tr	---	---	---	---
1638	1639	*cis*-Guaia-3,9-dien-11-ol	tr	tr	tr	---	tr	tr	---	tr	tr
1640	1638	τ-Cadinol	tr	tr	tr	---	tr	tr	---	tr	tr
1642	1640	τ-Muurolol	tr	tr	tr	---	tr	tr	---	tr	tr
1654	1652	α-Cadinol	0.1	0.1	0.1	0.1	0.1	0.1	0.1	0.1	0.1
1682	1683	Germacra-4(15),5,10(14)-trien-1α-ol	---	---	---	---	0.1	---	---	---	---
1688	1685	Eudesma-4(15),7-dien-1β-ol	---	---	---	---	0.1	---	---	---	---
1691	1688	Shyobunol	---	---	---	---	tr	---	---	---	---
1714	1715	Pentadecanal	---	---	---	---	---	---	---	tr	---
1765	1769	Benzyl benzoate	0.3	0.2	0.2	0.6	0.3	0.3	0.6	0.3	0.2
1840	1841	Phytone	---	---	---	---	tr	tr	---	tr	tr

2083	2081	1-Octadecanol	---	---	---	---	0.1	---	---	---	---

Supplementary Table S3. Essential oil composition of *Pycnanthemum virginianum* variety M3.

RI(calc)	RI(db)	Compound	M3-R1-H1	M3-R3-H1	M3-R1-H2	M3-R3-H3
844	841	3-Methylcyclopentanone	0.2	tr	---	---
925	925	α-Thujene	---	---	tr	tr
932	932	α-Pinene	---	tr	0.2	0.1
949	950	Camphene	---	tr	tr	tr
951	951	3-Methylcyclohexanone	tr	tr	tr	tr
952	953	Thuja-2,4(10)-diene	---	---	tr	tr
969	974	Hexanoic acid	0.1	---	---	---
972	972	Sabinene	---	tr	0.1	0.1
973	973	1-Octen-3-one	0.1	tr	---	---
977	978	β-Pinene	---	0.1	0.3	0.2
**979**	**978**	**1-Octen-3-ol**	**3.2**	**3.8**	**1.8**	**1.9**
984	983	3-Octanone	0.2	1.0	0.5	0.5
989	989	Myrcene	tr	0.2	0.4	0.4
997	996	3-Octanol	0.2	0.3	0.1	0.1
1004	1004	*p*-Mentha-1(7),8-diene	---	tr	tr	tr
1016	1017	α-Terpinene	---	---	---	tr
1024	1024	*p*-Cymene	0.3	0.1	tr	0.3
1029	1029	β-Phellandrene	tr	tr	---	---
1029	1030	Limonene	0.5	1.5	1.5	1.2
1031	1032	1,8-Cineole	0.1	0.1	0.3	0.3
1035	1034	(*Z*)-β-Ocimene	---	tr	tr	tr
1046	1050	3,4-Dimethylcyclopentan-1,2-dione	0.1	tr	---	---
1045	1045	(*E*)-β-Ocimene	---	0.1	0.1	0.2
1048	1058	3-Methylcyclohexane-1,2-dione	0.2	tr	---	---
1057	1057	γ-Terpinene	---	---	---	tr
1070	1069	*cis*-Sabinene hydrate	---	tr	tr	tr
1083	1088	3-Nonanone	---	tr	---	---
1082	1079	Non-1-en-3-ol	---	tr	---	tr
1085	1086	Terpinolene	---	---	tr	tr

1089	1093	*p*-Cymenene	---	---	---	tr
1101	1101	Linalool	---	---	tr	0.1
1105	1107	Nonanal	---	---	tr	tr
1107	1107	1-Octen-3-yl acetate	---	0.2	tr	tr
1124	1120	*trans*-*p*-Mentha-2,8-dien-1-ol	---	---	---	tr
1144	1140	*trans*-Sabinol	---	---	tr	0.1
1146	1144	*trans*-Tagetone	---	---	tr	---
1150	1149	*p*-Menth-3-en-8-ol	0.2	---	---	---
1150	1145	*trans*-Verbenol	0.1	0.1	0.1	0.1
**1156**	**1156**	**Menthone**	**4.6**	**26.0**	**3.8**	**5.6**
**1168**	**1166**	**Isomenthone**	**1.7**	**1.9**	**48.0**	**65.8**
1166	1164	Menthofuran	tr	tr	---	---
1173	1170	δ-Terpineol	---	---	tr	---
1175	1173	Borneol	---	---	tr	tr
1177	1176	*trans*-Isopulegone	1.7	1.4	1.2	1.1
1182	1183	Terpinen-4-ol	---	tr	tr	0.1
1184	1184	1-Decen-3-ol	---	tr	---	---
1186	1184	6-Methyl-2-vinyl-5-hepten-1-ol	---	---	tr	tr
1198	1195	α-Terpineol	0.2	0.2	0.3	0.3
1200	1201	*cis*-Piperitenol	---	---	---	0.1
1207	1208	Decanal	---	tr	tr	tr
1209	1208	Verbenone	0.3	0.1	0.1	0.1
1219	1221	3-Isopropylbenzaldehyde	---	---	0.2	0.2
1226	1230	Cuminaldehyde	---	0.1	---	---
**1242**	**1240**	**Pulegone**	**71.7**	**55.9**	**37.3**	**16.1**
1254	1254	Piperitone	---	tr	0.3	0.5
1258	1261	Pulegone oxide A	0.4	0.1	---	---
1268	1270	*iso*-Piperitenone	0.3	0.2	0.1	tr
1286	1287	Pulegone oxide B	5.0	0.5	0.2	0.1
1289	1289	Thymol	---	0.6	tr	0.6
1297	1300	Pulegone oxide C	0.2	tr	---	---

1297	1300	Carvacrol	---	tr	---	tr
1308	1309	4-Vinylguaiacol	---	---	---	tr
1331	1332	Bicycloelemene	---	---	---	tr
1335	1335	δ-Elemene	---	0.1	tr	0.2
1337	1339	Piperitenone	0.2	0.2	0.2	0.1
1338	---	Unidentified	0.2	---	---	0.3
1342	1348	Mint furanone isomer A	0.1	0.1	---	---
1344	1349	Mint furanone isomer B	0.1	tr	---	---
1375	1375	α-Copaene	---	tr	tr	tr
1381	1383	*cis*-β-Elemene	---	tr	tr	tr
1383	1382	β-Bourbonene	0.2	0.2	0.1	0.2
1385	1385	α-Bourbonene	---	---	---	tr
1389	1390	*trans*-β-Elemene	---	0.3	0.2	0.2
1391	1392	(*Z*)-Jasmone	0.2	0.1	0.1	0.1
1418	1417	(*E*)-β-Caryophyllene	---	1.5	1.0	1.1
1429	1430	β-Copaene	---	tr	tr	tr
1432	1432	*trans*-α-Bergamotene	0.1	0.2	0.2	0.1
1434	---	Unidentified	1.2	0.3	---	tr
1439	1440	(*Z*)-β-Farnesene	---	---	---	tr
1442	1447	*iso*-Germacrene D	---	---	---	tr
1452	1451	(*E*)-β-Farnesene	---	---	---	tr
1454	1454	α-Humulene	---	0.2	0.1	0.2
1466	1465	*cis*-Muurola-4(14),5-diene	---	---	---	tr
1473	1478	γ-Muurolene	---	tr	---	tr
1480	1480	Germacrene D	---	0.5	0.7	1.0
1483	1483	*trans*-β-Bergamotene	---	0.1	tr	tr
1484	1485	γ-Thujaplicin	0.2	0.1	---	---
1489	1489	(*Z*,*E*)-α-Farnesene	---	---	---	tr
1490	1493	Menthalactone	0.1	tr	---	---
1494	1497	Bicyclogermacrene	---	---	0.1	0.1
1497	1497	α-Muurolene	---	tr	---	tr

1503	1504	(*E*,*E*)-α-Farnesene	---	0.2	0.2	0.2
1506	1508	Germacrene A	---	---	tr	tr
1507	1508	β-Bisabolene	---	tr	tr	tr
1511	1512	γ-Cadinene	---	tr	---	tr
1517	1518	δ-Cadinene	---	tr	tr	tr
1523	1523	β-Sesquiphellandrene	---	0.1	0.1	0.1
1543	1546	α-Elemol	---	tr	---	---
1546	1546	α-Elemol	---	---	---	tr
1556	1557	Germacrene B	---	tr	---	tr
1559	1560	(*E*)-Nerolidol	---	tr	---	tr
1575	1576	Spathulenol	---	tr	tr	tr
1580	1577	Caryophyllene oxide	1.8	0.6	0.1	0.1
1607	1607	Humulene epoxide I	0.2	0.1	---	tr
1627	1629	*iso*-Spathulenol	0.1	0.2	0.1	tr
1637	1639	*cis*-Guaia-3,9-dien-11-ol	---	---	---	tr
1640	1638	τ-Cadinol	---	---	---	tr
1642	1640	τ-Muurolol	---	---	---	tr
1654	1655	α-Cadinol	---	0.1	tr	tr
1763	1769	Benzyl benzoate	---	---	---	tr

Supplementary Table S4. Essential oil composition of *Pycnanthemum virginianum* variety M4.

RI(calc)	RI(db)	Compound	M4-R1-H1	M4-R2-H1	M4-R3-H1	M4-R1-H2	M4-R2-H2	M4-R3-H2	M4-R1-H3	M4-R2-H3	M4-R3-H3
925	925	α-Thujene	tr	0.2	1.8	0.1	0.5	1.0	0.1	0.1	0.2
932	932	α-Pinene	tr	0.1	0.6	0.1	0.2	0.4	tr	0.1	0.1
949	950	Camphene	tr	tr	0.2	0.1	0.2	0.3	tr	0.1	0.2
952	951	3-Methylcyclohexanone	---	---	---	---	---	---	tr	---	---
952	953	Thuja-2,4(10)-diene	---	---	---	---	tr	tr	---	tr	tr
962	960	Benzaldehyde	---	---	---	---	tr	---	tr	tr	tr
970	971	Verbenene	---	---	---	---	tr	---	---	---	---
972	971	Sabinene	tr	0.1	0.2	tr	0.1	0.1	tr	tr	tr
973	973	1-Octen-3-one	---	---	---	---	---	---	---	tr	---
977	978	β-Pinene	tr	0.1	0.4	0.1	0.3	0.3	0.1	0.1	0.2
**979**	**978**	**1-Octen-3-ol**	**3.7**	**2.7**	**3.1**	**5.5**	**6.1**	**4.8**	**4.0**	**6.3**	**7.0**
984	983	3-Octanone	0.4	0.3	0.4	0.8	0.9	0.7	0.6	0.7	0.8
989	989	Myrcene	0.7	0.9	2.4	0.3	0.9	1.2	0.6	0.4	0.5
997	996	3-Octanol	0.1	0.1	0.1	0.2	0.2	0.1	0.1	0.2	0.2
1004	1004	*p*-Mentha-1(7),8-diene	---	tr	---	---	tr	tr	---	tr	---
1007	1007	α-Phellandrene	0.1	0.1	0.3	tr	0.1	0.1	0.1	tr	tr
1009	1008	δ-3-Carene	tr	tr	0.1	tr	0.1	0.1	tr	tr	tr
1017	1017	α-Terpinene	0.7	0.9	2.8	0.2	0.9	1.2	0.6	0.3	0.3
1020	1022	*m*-Cymene	tr	tr	---	0.1	0.1	tr	tr	tr	0.1
**1025**	**1024**	***p*-Cymene**	**3.5**	**3.3**	**8.1**	**19.7**	**24.1**	**23.7**	**10.5**	**18.2**	**28.1**
1027	1026	2-Acetyl-3-methylfuran	---	tr	---	0.8	0.6	0.5	0.3	1.0	1.1
1029	1030	Limonene	0.3	0.9	0.8	0.5	0.7	0.8	0.5	0.6	0.4
1030	1029	β-Phellandrene	0.1	0.1	0.2	0.1	0.2	0.2	0.1	0.1	0.1
1031	1030	1,8-Cineole	0.1	0.1	tr	0.1	0.1	0.1	0.1	0.2	0.2
1035	1034	(*Z*)-β-Ocimene	0.2	0.2	0.4	tr	0.2	0.2	0.2	0.1	0.2
1045	1045	(*E*)-β-Ocimene	1.5	1.0	2.3	---	0.1	0.2	0.2	0.2	0.2
1057	1057	γ-Terpinene	2.4	2.6	6.7	tr	1.4	2.1	1.5	1.0	1.0
1069	1069	*cis*-Sabinene hydrate	1.2	0.7	1.0	3.4	2.3	1.9	1.5	3.1	3.4
1080	1079	1-Nonen-3-ol	0.1	tr	tr	0.1	0.1	0.1	0.1	0.1	0.1

1085	1086	Terpinolene	0.1	0.1	0.1	0.1	0.2	0.1	0.1	0.1	0.1
1090	1091	*p*-Cymenene	---	---	tr	0.2	0.2	0.1	0.1	0.3	0.4
1100	1099	Linalool	0.3	0.2	0.2	0.3	0.4	0.3	0.3	0.3	0.2
1101	1099	*trans*-Sabinene hydrate	0.1	0.1	0.1	0.7	0.5	0.4	0.3	0.8	0.8
1107	1107	1-Octen-3-yl acetate	0.1	0.1	0.2	0.6	0.6	0.5	0.5	0.6	0.8
1113	---	2,4-Dimethylhepta-2,4-dienal	---	---	---	0.1	0.2	0.1	0.1	0.2	0.2
1117	---	2,4-Dimethylhepta-2,4-dienal	---	---	---	0.2	0.2	0.1	0.1	0.2	0.3
1121	1121	*trans*-*p*-Mentha-2,8-dien-1-ol	---	---	---	0.1	0.1	tr	tr	0.1	0.1
1124	1124	*cis*-*p*-Menth-2-en-1-ol	---	---	---	0.1	0.1	0.1	tr	0.1	0.1
1132	1132	*cis*-Limonene oxide	---	---	---	0.1	0.1	tr	tr	0.1	tr
1137	1138	*trans*-Limonene oxide	---	---	---	0.1	0.2	0.1	0.1	0.2	0.1
1139	1140	*trans*-Sabinol	---	---	---	---	0.1	tr	---	0.1	tr
1140	1140	*trans*-Pinocarveol	---	---	---	0.1	0.2	0.1	0.1	0.2	0.2
1142	1142	*trans*-*p*-Menth-2-en-1-ol	---	---	---	---	0.1	0.1	0.1	0.1	---
1145	1145	*trans*-Verbenol	---	---	---	0.3	0.5	0.2	0.2	0.6	0.7
1147	1145	Camphor	---	---	---	---	---	---	---	0.1	---
1150	1150	α-Phellandren-8-ol	---	---	---	---	tr	---	---	---	---
1150	1150	*p*-Menth-3-en-8-ol	---	---	---	---	---	---	0.1	tr	---
1156	1156	Menthone	0.1	0.6	tr	tr	0.1	tr	1.1	1.4	0.1
**1165**	**1166**	**Isomenthone**	**1.5**	**6.3**	**0.4**	**tr**	**1.0**	**tr**	**12.4**	**5.9**	**0.7**
1169	1168	*trans*-Phellandrene epoxide	---	---	---	---	0.1	---	---	0.1	0.1
1171	1170	Borneol	0.4	0.2	0.3	0.9	1.6	0.7	0.5	1.4	1.3
1176	1176	*trans*-Isopulegone	---	0.7	---	---	---	---	0.2	0.5	---
1180	1180	Terpinen-4-ol	0.6	0.4	0.7	1.1	2.0	1.1	1.0	1.7	1.6
1188	1188	*p*-Cymen-8-ol	---	---	0.1	1.7	1.6	0.9	0.7	2.6	3.2
1195	1195	α-Terpineol	0.1	0.2	0.1	0.2	0.3	---	0.2	0.3	0.2
1200	1201	*cis*-Piperitenol	---	0.1	---	---	---	---	---	0.3	---
1209	1208	Verbenone	---	---	---	---	---	0.2	---	0.1	---
1215	1216	3-Isopropylbenzaldehyde	0.1	tr	tr	0.2	0.1	---	0.3	0.1	0.1
1215	1217	Coumaran	---	---	---	---	---	0.1	---	---	0.1
1221	1223	*trans*-Carveol	---	---	---	---	0.1	1.6	---	0.2	0.1

1229	1229	Thymol methyl ether	0.4	0.3	0.2	1.6	2.4	---	5.4	3.9	4.5
1232	1232	*cis*-Carveol	---	---	---	---	---	tr	---	0.1	---
**1241**	**1241**	**Pulegone**	**4.2**	**29.5**	**1.6**	**0.1**	**1.7**	**tr**	**4.0**	**2.5**	**0.3**
1242	1242	Cuminal	---	---	---	0.2	---	---	---	0.5	0.5
1243	1242	Carvone	---	---	---	---	---	---	---	0.1	---
1250	1252	Thymoquinone	---	---	---	11.0	1.7	0.8	0.9	1.0	1.0
1255	1254	Piperitone	---	---	---	---	---	---	0.9	0.1	---
**1290**	**1289**	**Thymol**	**68.6**	**38.3**	**54.6**	**34.0**	**32.7**	**42.6**	**37.1**	**28.1**	**27.6**
1293	1287	Pulegone oxide B	---	---	---	---	---	---	---	0.1	---
1294	1291	*p*-Cymen-7-ol	---	---	---	0.8	0.8	0.5	0.2	1.2	1.2
1299	1296	Carvacrol	1.7	0.9	1.2	1.4	0.8	1.1	0.7	0.8	0.6
1300	1299	Perilla alcohol	---	---	---	---	tr	---	---	0.1	---
1305	1306	*iso*-Ascaridol	---	---	---	0.1	tr	tr	---	0.1	0.1
1309	1309	4-Vinylguaiacol	---	---	---	0.1	0.1	0.1	0.1	0.1	0.1
1331	1326	Bicycloelemene	---	0.1	0.1	---	tr	tr	0.1	---	---
1335	1335	δ-Elemene	0.1	0.5	0.5	0.2	0.2	0.2	0.4	0.2	0.1
1339	1339	Piperitenone	tr	0.1	---	---	---	---	0.1	---	---
1340	---	Unidentified	0.1	0.4	---	---	---	---	0.2	1.2	0.2
1341	1345	Thymyl acetate	---	---	0.1	---	---	---	---	---	---
1375	1375	α-Copaene	0.1	0.1	0.1	0.1	0.1	0.1	0.1	0.1	0.1
1381	1383	*cis*-β-Elemene	---	tr	---	---	---	---	tr	---	---
1382	1382	β-Bourbonene	0.1	0.1	0.1	0.3	0.2	0.2	0.4	0.2	0.3
1383	1385	(*E*)-Jasmone	---	---	---	---	0.1	---	---	0.1	---
1389	1390	*trans*-β-Elemene	0.2	0.3	0.3	0.3	0.3	0.2	0.4	0.3	0.2
1391	1392	(*Z*)-Jasmone	0.1	0.1	0.1	0.1	0.1	0.1	0.1	0.1	0.1
1403	1405	(*Z*)-β-Caryophyllene	---	---	---	0.1	0.1	tr	tr	tr	0.1
1417	1422	β-Ylangene	---	---	---	---	---	---	---	---	0.1
1418	1417	(*E*)-β-Caryophyllene	1.7	1.6	2.0	2.1	1.7	1.4	2.3	1.4	0.9
1429	1430	β-Copaene	0.1	0.1	0.1	0.2	0.1	0.1	0.1	0.1	0.1
1432	1432	*trans*-α-Bergamotene	0.3	0.3	0.3	0.4	0.2	0.2	0.4	0.3	0.2
1454	1453	α-Humulene	0.3	0.3	0.4	0.3	0.3	0.2	0.4	0.2	0.2

1474	1478	γ-Muurolene	0.1	tr	0.1	0.1	0.1	0.1	0.1	0.1	0.1
1476	1475	γ-Gurjunene	0.1	tr	0.1	0.2	0.1	0.1	0.1	0.1	0.1
1480	1480	Germacrene D	0.8	1.8	1.5	0.8	0.8	0.8	1.7	0.8	0.5
1483	1483	*trans*-β-Bergamotene	0.1	0.1	0.1	0.1	0.1	0.1	0.1	0.1	0.1
1485	1485	γ-Thujaplicin	---	---	---	1.1	0.2	0.1	0.1	0.2	0.1
1487	1489	δ-Selinene	tr	tr	tr	---	---	---	---	---	---
1487	1489	β-Selinene	0.1	tr	0.1	0.2	0.1	0.1	0.1	0.2	0.1
1490	1493	Callicarpenal	---	---	---	0.2	0.1	0.1	0.1	0.2	0.2
1491	1491	Viridiflorene	tr	tr	tr	---	---	---	---	---	---
1492	1490	γ-Amorphene	tr	tr	tr	---	---	---	---	---	---
1494	1497	Bicyclogermacrene	---	0.4	0.3	0.1	0.2	0.2	0.3	0.2	0.1
1494	1497	α-Selinene	0.2	---	0.1	0.1	---	---	---	---	0.1
1497	1497	α-Muurolene	tr	tr	tr	0.1	tr	tr	tr	tr	tr
1503	1504	(*E*,*E*)-α-Farnesene	0.5	0.4	0.4	---	---	tr	0.1	0.1	0.1
1506	1508	β-Bisabolene	0.1	0.1	0.1	0.1	tr	tr	0.1	0.1	tr
1512	1512	γ-Cadinene	0.1	tr	0.1	0.1	0.1	tr	tr	0.1	0.1
1513	1515	Cubebol	---	---	---	---	tr	---	---	tr	---
1517	1518	δ-Cadinene	0.2	0.1	0.2	0.1	0.1	0.1	0.1	0.1	0.1
1523	1523	β-Sesquiphellandrene	0.3	0.3	0.3	0.4	0.2	0.2	0.4	0.2	0.2
1536	1538	α-Cadinene	tr	---	---	---	---	---	---	---	---
1546	1546	α-Elemol	tr	tr	tr	---	tr	tr	---	0.1	tr
1551	1549	Thymohydroquinone	---	0.1	0.2	0.6	1.0	1.4	1.0	0.1	0.1
1557	1557	Germacrene B	tr	tr	tr	---	---	tr	---	---	---
1560	1560	(*E*)-Nerolidol	tr	tr	tr	---	---	tr	---	---	---
1575	1576	Spathulenol	0.3	0.1	0.1	0.4	0.3	0.3	0.3	0.4	0.5
1580	1577	Caryophyllene oxide	0.3	0.1	0.1	1.0	0.9	0.6	0.6	1.2	1.6
1607	1607	Humulene epoxide I	tr	---	---	0.2	0.1	0.1	0.1	0.2	0.2
1627	1629	*iso*-Spathulenol	0.3	0.1	0.2	0.5	0.4	0.3	0.3	0.4	0.4
1631	1630	Caryophylla-4(12),8(13)-dien-5α-ol	---	---	---	---	0.1	---	---	0.1	---
1635	1635	Caryophylla-4(12),8(13)-dien-5β-ol	---	---	---	---	0.1	tr	---	0.1	0.1
1636	1638	τ-Cadinol	tr	tr	tr	---	---	---	---	---	---

1638	1640	τ-Muurolol	tr	tr	tr	---	---	---	---	---	---
1639	1639	*cis*-Guaia-3,9-dien-11-ol	0.1	0.1	0.1	---	---	---	---	---	---
1654	1652	α-Cadinol	0.1	0.1	0.1	---	0.1	0.1	0.1	0.1	0.1
1658	1658	Selin-11-en-4α-ol	tr	tr	tr	---	---	---	---	---	---
1764	1769	Benzyl benzoate	---	0.1	---	---	---	---	tr	---	---
1840	1841	Phytone	---	---	---	---	---	---	tr	---	---

Supplementary Table S5. Enantiomeric distribution of terpenoid constituents of *Pycnanthemum virginianum*, variety M1.

	Enantiomeric Distribution (ED), (+)-enantiomer : (-)-enantiomer								
Compound	M1-R1-H1	M1-R2-H1	M1-R3-H1	M1-R1-H2	M1-R2-H2	M1-R3-H2	M1-R1-H3	M1-R2-H3	M1-R3-H3
α-Thujene	53.8:46.2	53.0:47.0	50.3:49.7	---	---	---	---	---	---
α-Pinene	25.8:74.2	25.2:74.8	24.3:75.7	24.2:75.8	22.4:77.6	24.2:75.8	23.7:76.3	24.3:75.7	24.5:75.5
Camphene	100:0	100:0	100:0	---	---	---	---	---	---
Sabinene	33.1:66.9	34.8:65.2	33.9:66.1	28.6:71.4	26.2:73.8	28.9:71.1	29.2:70.8	27.9:72.1	28.4:71.6
β-Pinene	47.3:52.7	46.2:53.8	47.0:53.0	46.4:53.6	45.6:54.4	46.5:53.5	45.5:54.5	46.3:53.7	46.1:53.9
Limonene	6.2:93.8	6.0:94.0	6.7:93.3	6.7:93.3	7.5:92.5	6.8:93.2	0:100	6.8:93.2	7.1:92.9
Menthone	0:100	0:100	0:100	0:100	0:100	0:100	0:100	0:100	0:100
Isomenthone	100:0	100:0	100:0	100:0	100:0	100:0	100:0	100:0	100:0
α-Terpineol	9.5:90.5	9.7:90.3	10.6:89.4	7.8:92.2	9.3:90.7	8.8:91.2	9.2:90.8	9.6:90.4	8.1:91.9
Pulegone	100:0	100:0	100:0	100:0	100:0	100:0	100:0	100:0	100:0
Piperitone	100:0	100:0	100:0	91.5:8.5	91.0:9.0	91.6:8.4	91.0:9.0	91.2:8.8	91.2:8.8
trans-β-Elemene	16.5:83.5	16.9:83.1	16.3:83.7	15.8:84.2	14.8:85.2	14.3:85.7	15.0:85.0	13.6:86.4	14.5:85.5
(*E*)-β-Caryophyllene	100:0	100:0	100:0	100:0	100:0	100:0	100:0	100:0	100:0
Germacrene D	90.0:10.0	92.5:7.5	90.8:9.2	91.4:8.6	91.1:8.9	90.8:9.2	91.2:8.8	90.3:9.7	90.2:9.8
δ-Cadinene	0:100	0:100	0:100	0:100	0:100	0:100	0:100	0:100	0:100

Supplementary Table S6. Enantiomeric distribution of terpenoid constituents of *Pycnanthemum virginianum*, variety M2.

	Enantiomeric Distribution (ED), (+)-enantiomer : (-)-enantiomer								
Compound	M2-R1-H1	M2-R2-H1	M2-R3-H1	M2-R1-H2	M2-R2-H2	M2-R3-H2	M2-R1-H3	M2-R2-H3	M2-R3-H3
α-Thujene	72.3:27.7	72.4:27.6	72.2:27.8	72.6:27.4	71.9:28.1	72.1:27.9	69.1:30.9	71.9:28.1	71.9:28.1
α-Pinene	57.9:42.1	57.5:42.5	56.6:43.4	60.4:39.6	59.8:40.2	57.6:42.4	---	57.8:42.2	55.7:44.3
Camphene	100:0	100:0	100:0	100:0	100:0	100:0	---	100:0	100:0
Sabinene	0:100	0:100	0:100	0:100	0:100	0:100	0:100	0:100	0:100
β-Pinene	55.0:45.0	54.1:45.9	54.1:45.9	56.2:43.8	55.4:44.6	54.3:45.7	54.4:45.6	54.5:45.5	52.3:47.7
α-Phellandrene	93.6:6.4	95.4:4.6	95.5:4.5	95.0:5.0	100:0	94.8:5.2	---	93.6:6.4	95.5:4.5
δ-3-Carene	100:0	100:0	100:0	100:0	100:0	100:0	---	100:0	100:0
α-Terpinene	100:0	100:0	100:0	100:0	100:0	100:0	100:0	100:0	100:0
Limonene	0:100	0:100	0:100	0:100	0:100	0:100	0.2:99.8	0:100	0:100
β-Phellandrene	0:100	0:100	0:100	0:100	0:100	0:100	0:100	0:100	0:100
cis-Sabinene hydrate	94.3:6.7	95.7:4.3	94.2:5.8	96.2:3.8	95.1:4.9	94.3:5.7	95.7:4.3	95.1:4.9	93.7:6.3
Linalool	100:0	100:0	100:0	100:0	100:0	100:0	100:0	100:0	100:0
trans-Sabinene hydrate	80.5:19.5	78.2:21.8	78.6:21.4	80.5:19.5	79.6:20.4	78.2:21.8	79.2:20.8	75.5:24.5	75.6:25.4
Menthone	0:100	0:100	0:100	0:100	0:100	0:100	0:100	0:100	0:100
Isomenthone	---	---	---	100:0	100:0	100:0	100:0	100:0	100:0
Borneol	0:100	0:100	0:100	0:100	0:100	0:100	0:100	0:100	0:100
Terpinen-4-ol	64.3:35.7	64.2:35.8	64.5:35.5	65.6:34.4	64.7:35.3	64.7:35.3	65.0:35.0	63.8:36.2	63.9:36.1
α-Terpineol	32.5:67.5	31.8:68.2	31.7:68.3	31.9:68.1	31.7:68.3	31.8:68.2	31.6:68.4	32.2:67.8	33.2:66.8
Pulegone	100:0	100:0	100:0	100:0	100:0	100:0	100:0	100:0	100:0
trans-β-Elemene	22.4:77.6	22.1:77.9	22.2:77.8	19.5:80.5	20.1:79.9	20.4:79.6	12.8:87.2	20.8:79.2	20.3:79.7
(*E*)-β-Caryophyllene	100:0	100:0	100:0	100:0	100:0	100:0	100:0	100:0	100:0
Germacrene D	94.3:5.7	95.1:4.9	94.2:5.8	93.8:6.2	94.7:5.3	94.3:5.7	93.8:6.2	94.5:5.5	94.5:5.5
δ-Cadinene	0:100	0:100	0:100	0:100	0:100	0:100	0:100	0:100	0:100

Supplementary Table S7. Enantiomeric distribution of terpenoid constituents of *Pycnanthemum virginianum*, variety M3.

	ED, (+):(-)			
Compound	M3-R1-H1	M3-R3-H1	M3-R1-H2	M3-R3-H3
α-Pinene	---	21.1:78.9	24.3:75.7	24.8:75.2
Sabinene	---	---	27.7:72.3	28.4:71.6
β-Pinene	---	46.7:53.3	45.9:54.1	45.0:55.0
Limonene	7.7:92.3	6.2:93.8	6.9:93.1	6.8:93.2
Menthone	0:100	0:100	0:100	0:100
Isomenthone	100:0	100:0	100:0	100:0
α-Terpineol	10.8:89.2	10.7:89.3	8.7:91.3	8.7:91.3
Pulegone	100:0	100:0	100:0	100:0
Piperitone	---	85.9:14.1	90.2:9.8	92.0:8.0
trans-β-Elemene	---	18.0:82.0	15.9:84.1	14.3:85.7
(*E*)-β-Caryophyllene	---	100:0	100:0	100:0
Germacrene D	---	92.5:7.5	91.2:8.8	90.0:9.1
δ-Cadinene	---	0:100	0:100	0:100

Supplementary Table S8. Enantiomeric distribution of terpenoid constituents of *Pycnanthemum virginianum*, variety M4.

	Enantiomeric Distribution (ED), (+)-enantiomer : (-)-enantiomer								
Compound	M4-R1-H1	M4-R2-H1	M4-R3-H1	M4-R1-H2	M4-R2-H2	M4-R3-H2	M4-R1-H3	M4-R2-H3	M4-R3-H3
α-Thujene	72.1:27.9	76.3:23.7	77.2:22.8	73.1:26.9	75.7:24.3	76.6:23.4	76.4:23.6	78.2:21.8	73.8:26.2
α-Pinene	66.3:33.7	65.7:34.3	74.2:25.8	73.5:26.5	74.3:25.7	74.8:25.2	69.6:30.4	68.9:31.1	73.4:26.6
Camphene	100:0	100:0	100:0	100:0	100:0	100:0	100:0	100:0	100:0
Sabinene	48.0:52.0	40.7:59.3	0:100	---	71.9:28.1	67.3:32.7	---	---	67.1:32.9
β-Pinene	26.9:73.1	32.0:68.0	32.5:67.6	42.2:57.8	33.4:66.6	31.1:68.9	24.4:75.6	27.8:72.2	28.1:71.9
α-Phellandrene	93.7:6.3	95.3:4.7	95.1:4.9	100:0	93.7:6.3	92.6:7.4	93.6:6.4	100:0	100:0
δ-3-Carene	100:0	100:0	100:0	100:0	100:0	100:0	100:0	100:0	100:0
α-Terpinene	100:0	100:0	100:0	100:0	100:0	100:0	100:0	100:0	100:0
Limonene	0:100	0:100	0:100	49.0:50.1	0:100	2.1:97.9	0:100	12.3:87.7	15.2:84.8
β-Phellandrene	0:100	0:100	0:100	0:100	0:100	0:100	0:100	0:100	0:100
cis-Sabinene hydrate	98.4:1.6	98.1:1.9	98.4:1.6	98.5:1.5	98.5:1.5	98.7:1.3	98.6:1.4	98.6:1.4	98.6:1.4
Linalool	100:0	100:0	100:0	21.8:78.2	100:0	18.7:81.3	100:0	100:0	100:0
trans-Sabinene hydrate	81.4:18.6	81.0:19.0	81.5:18.5	81.2:18.8	82.5:17.5	82.6:17.4	82.7:17.3	82.9:17.1	82.1:17.9
Menthone	0:100	0:100	0:100	---	0:100	---	0:100	0:100	0:100
Isomenthone	100:0	100:0	100:0	---	100:0	---	100:0	100:0	100:0
Borneol	0:100	0:100	0:100	0:100	0:100	0:100	0:100	0:100	0:100
Terpinen-4-ol	59.9:40.1	63.0:37.0	64.7:35.3	63.4:36.6	67.7:32.3	63.7:36.3	71.8:28.2	68.2:31.8	67.6:32.4
α-Terpineol	73.1:26.9	32.6:67.4	73.0:27.0	100:0	100:0	100:0	100:0	100:0	100:0
Pulegone	100:0	100:0	100:0	100:0	100:0	---	100:0	100:0	100:0
δ-Elemene	0:100	50.2:49.8	0:100	0:100	0:100	0:100	48.8:51.2	---	---
α-Copaene	100:0	100:0	100:0	100:0	100:0	100:0	100:0	100:0	100:0
trans-β-Elemene	13.5:86.5	13.1:86.9	12.0:88.0	0:100	0:100	3.7:96.3	9.2:90.8	8.0:92.0	0:100
(*E*)-β-Caryophyllene	100:0	100:0	100:0	100:0	100:0	100:0	100:0	100:0	100:0
Germacrene D	84.3:15.7	84.6:15.4	81.4:18.6	77.2:22.8	77.0:23.0	76.6:23.4	84.5:15.5	83.4:16.6	78.7:21.3
δ-Cadinene	0:100	0:100	0:100	0:100	0:100	0:100	0:100	0:100	0:100
