# Supplementary figures and images for: Essential Oils of Four Virginia Mountain Mint (Pycnanthemum virginianum) Varieties Grown in North Alabama
Source: Plants (Basel). 2021 Jul 8;10(7):1397. doi: 10.3390/plants10071397 (PMC8309247; doi:10.3390/plants10071397)

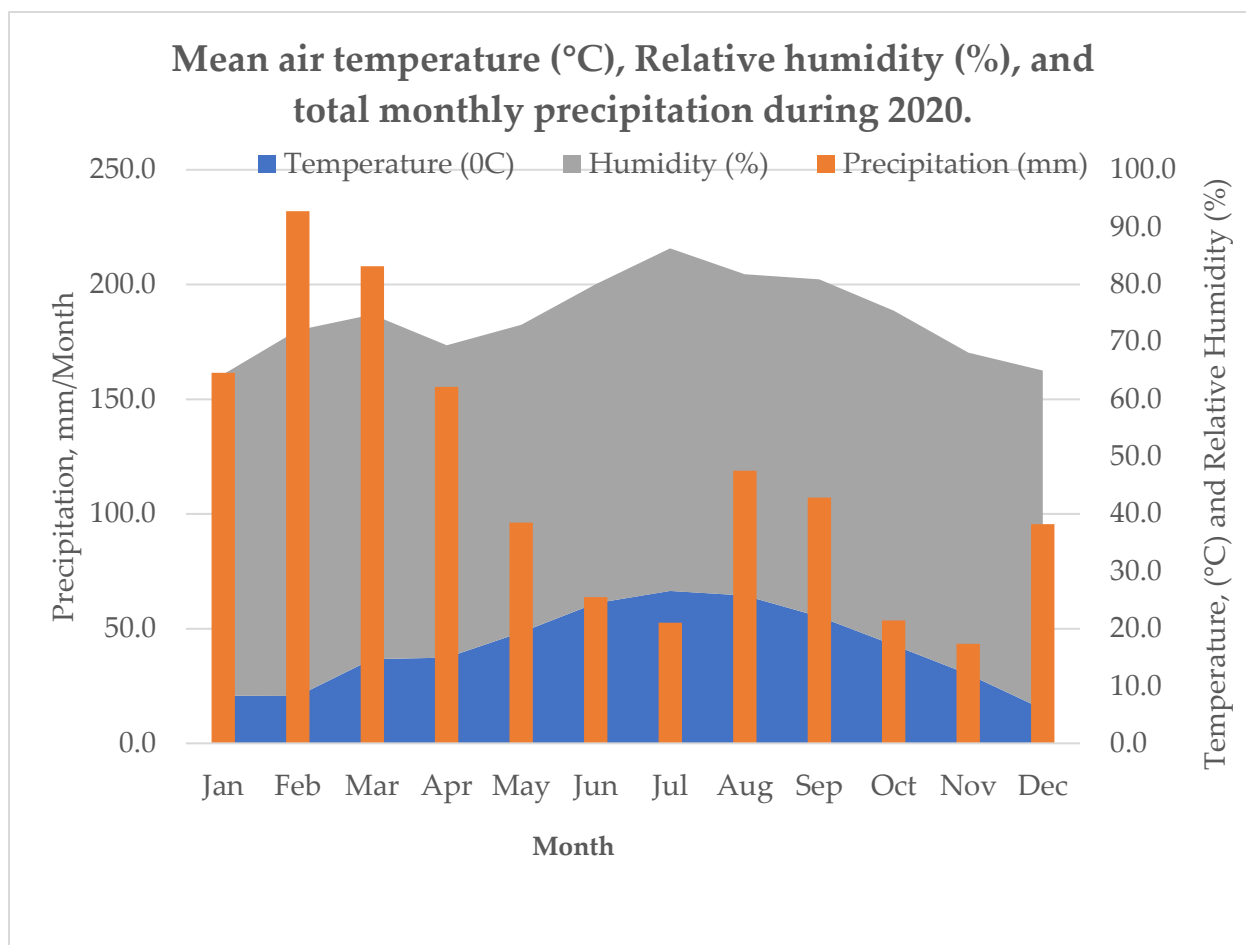

**Figure S1.** Huntsville, Alabama, weather data, 2020.

Supplement: Supplementary file 1 [file plants-10-01397-s001.zip › plants-1268847_Supplementary_Figure_S1.pdf]
